# Unexpected organic hydrate luminogens in the solid state

**DOI:** 10.1038/s41467-021-22685-0

**Published:** 2021-04-20

**Authors:** Feng Zhou, Peiyang Gu, Zhipu Luo, Hari Krishna Bisoyi, Yujin Ji, Youyong Li, Qingfeng Xu, Quan Li, Jianmei Lu

**Affiliations:** 1 College of Chemistry, Chemical Engineering and Materials Science, Collaborative Innovation Center of Suzhou Nano Science and Technology, Soochow University, Suzhou, Jiangsu 215123 China; 2 Institute of Molecular Enzymology, School of Biology and Basic Medical Sciences, Soochow University, Suzhou, China; 3 Advanced Materials and Liquid Crystal Institute and Chemical Physics Interdisciplinary Program, Kent State University, Kent, OH USA; 4 Institute of Functional Nano & Soft Materials (FUNSOM), Jiangsu Key Laboratory for Carbon-Based Functional Materials & Devices, Soochow University, Suzhou, Jiangsu China; 5 Institute of Advanced Materials and School of Chemistry and Chemical Engineering, Southeast University, Nanjing, Jiangsu China

**Keywords:** Optical materials, Synthesis and processing, Organic molecules in materials science

## Abstract

Developing organic photoluminescent materials with high emission efficiencies in the solid state under a water atmosphere is important for practical applications. Herein, we report the formation of both intra- and intermolecular hydrogen bonds in three tautomerizable Schiff-base molecules which comprise active hydrogen atoms that act as proton donors and acceptors, simultaneously hindering emission properties. The intercalation of water molecules into their crystal lattices leads to structural rearrangement and organic hydrate luminogen formation in the crystalline phase, triggering significantly enhanced fluorescence emission. By suppressing hydrogen atom shuttling between two nitrogen atoms in the benzimidazole ring, water molecules act as hydrogen bond donors to alter the electronic transition of the molecular keto form from nπ* to lower-energy ππ* in the excited state, leading to enhancing emission from the keto form. Furthermore, the keto-state emission can be enhanced using deuterium oxide (D_2_O) owing to isotope effects, providing a new opportunity for detecting and quantifying D_2_O.

## Abstract (web summary)

Developing organic photoluminescent materials with high emission efficiencies in the solid state under a water atmosphere is important for practical applications. Here, the authors report the formation of intra- and intermolecular hydrogen bonds in a tautomerizable Schiff base and intercalation of water in the crystal lattice leading to a luminescent organic hydrate.

## Introduction

Water is essential for regulating physiological and biochemical processes in living organisms^1–5^. Water can form intermolecular hydrogen bonds with inorganic compounds, organic molecules, and biological macromolecules, such as proteins and enzymes, inducing fascinating functionalities^6–13^. For example, water can help remove damaged adenine residues from oligonucleotides in the presence of a DNA glycosylase^14^. The structural participation of bound water in biomolecular recognition provides effective predictions for drug design^15^. However, water also causes some problems and limits the application of certain devices, such as perovskite solar cells and organic light-emitting diodes (OLEDs). For example, the performance of perovskite solar cells under an air atmosphere dramatically decreases over time because water in the air destroys the perovskite structure. Hydrogen bonding is among the most important noncovalent intermolecular interactions^16^, determining molecular conformation, molecular aggregation, and the function of numerous macroscopic substances. For example, bound water molecules exhibit liquid properties rather than gas properties owing to the existence of multiple intermolecular hydrogen bonds. In addition, hydrogen bond can also be used to design smart materials, such as self-healing polymers^17,18^, artificial muscle^19^, and organic or inorganic fluorescent/phosphorescent materials^20,21^. Hydrogen bond-assisted emission behaviour has mostly been attributed to restricted intramolecular rotation and vibration^22,23^.

Smart (stimuli-responsive) organic materials are able to reversibly respond to external stimuli, allowing their properties to be controlled by environmental stimuli, including temperature, electricity, mechanical, magnetic fields, chemicals, and stress^24–28^. Organic photoluminescent (PL) materials, as a class of smart organic materials, have gained considerable attention owing to their highly sensitive signal response and practicality-no sophisticated or expensive instruments are required^29–34^. However, in polar solvents, such as water, many organic PL materials show weak or quenched luminescence owing to polar interactions between the electronic structure and water, limiting their practical applications. Recently, certain organic PL materials have shown strong luminescence in the solid state without any solvent. In contrast, the emission behaviour of organic PL materials in the solid state under a water atmosphere remains in its infancy, but is highly important because most practical applications, such as OLEDs, are performed under an air atmosphere. Therefore, we are interested in designing a type of molecule to achieve high emission efficiencies in the solid state under a water atmosphere as an important development in this field.

**Fig. 1:**
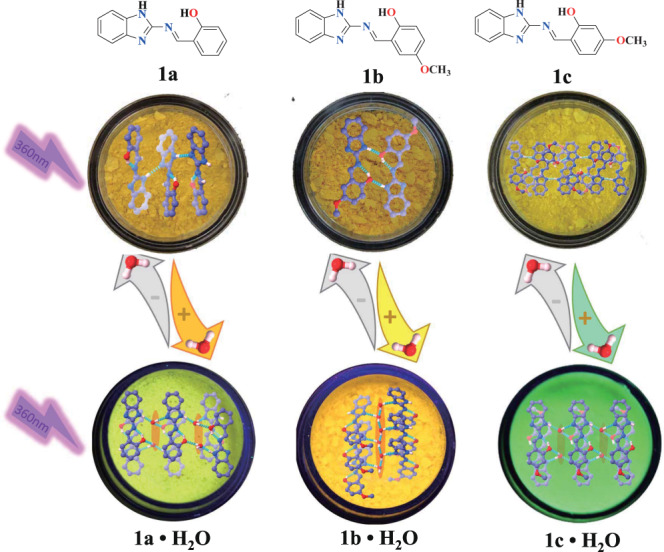
Schematic of turn-on molecular emission. Water-induced in situ changes in molecular organisation and turn-on of molecular emission for polycrystalline powders of **1a**, **1b**, and **1c**. Intercalation of water molecules into crystal lattices was observed to alter the surroundings of these molecules, leading to structural rearrangement in the crystalline state. These structural changes triggered significantly enhanced fluorescence emission.

Herein, we report a series of organic PL materials that can form hydrated crystals with water to produce organic hydrate luminogens (OHLs; Fig. 1, Supplementary Figs. 1, 5, 9, and 13). These OHLs are tautomerizable Schiff-base materials (2-(((1H-benzo[*d*]imidazol-2-yl)imino)methyl)-4-methoxyphenol (named as **1a**), 2-(((1H-benzo[*d*]imidazol-2-yl)imino)methyl)phenol (named as **1b**), and 2-(((1H-benzo[*d*]imidazol-2-yl)imino)methyl)-5-methoxyphenol (named as **1c**)) in which water plays a crucial role in regulating molecular stacking modes and effectively induces turn-on fluorescence. Hydrated single crystals (microcrystals) and anhydrous single crystals of the compounds were obtained in the presence and absence, respectively, of water (H_2_O) or deuterium oxide (D_2_O). The three Schiff-base compounds in anhydrous organic solvents, the anhydrous polycrystalline powder state, and the anhydrous single crystal state all showed weak fluorescence. However, their water-containing microcrystals and single hydrate crystals exhibited enhanced fluorescence due to the formation of a hydrogen-bonding network, which hindered excited state tautomerization and thus minimized energy dissipation. Intercalated water molecules altered the molecular reorganization of these small organic compounds in the hydrated crystalline state. Strong affinity between organic molecules and water blocked the in situ structural rearrangement due to the formation of intermolecular hydrogen bonds in the hydrated crystals. Furthermore, reversible “on-off” fluorescence switching of the three organic compounds was achieved by hydration and dehydration of their polycrystalline samples. Compounds **1a**, **1b**, and **1c** in anhydrous polycrystalline and crystalline phases induced strong light emission through in situ assembly with water (Fig. 1). The mechanism of enhanced luminescence in these compounds was attributed to the inhibited tautomer formation of benzimidazoles, which altered the nπ* to ππ* electronic transition of their keto form, and increased molecular rigidity of their assemblies in the solid state.

## Results

### Synthetic target compounds

**Fig. 2:**
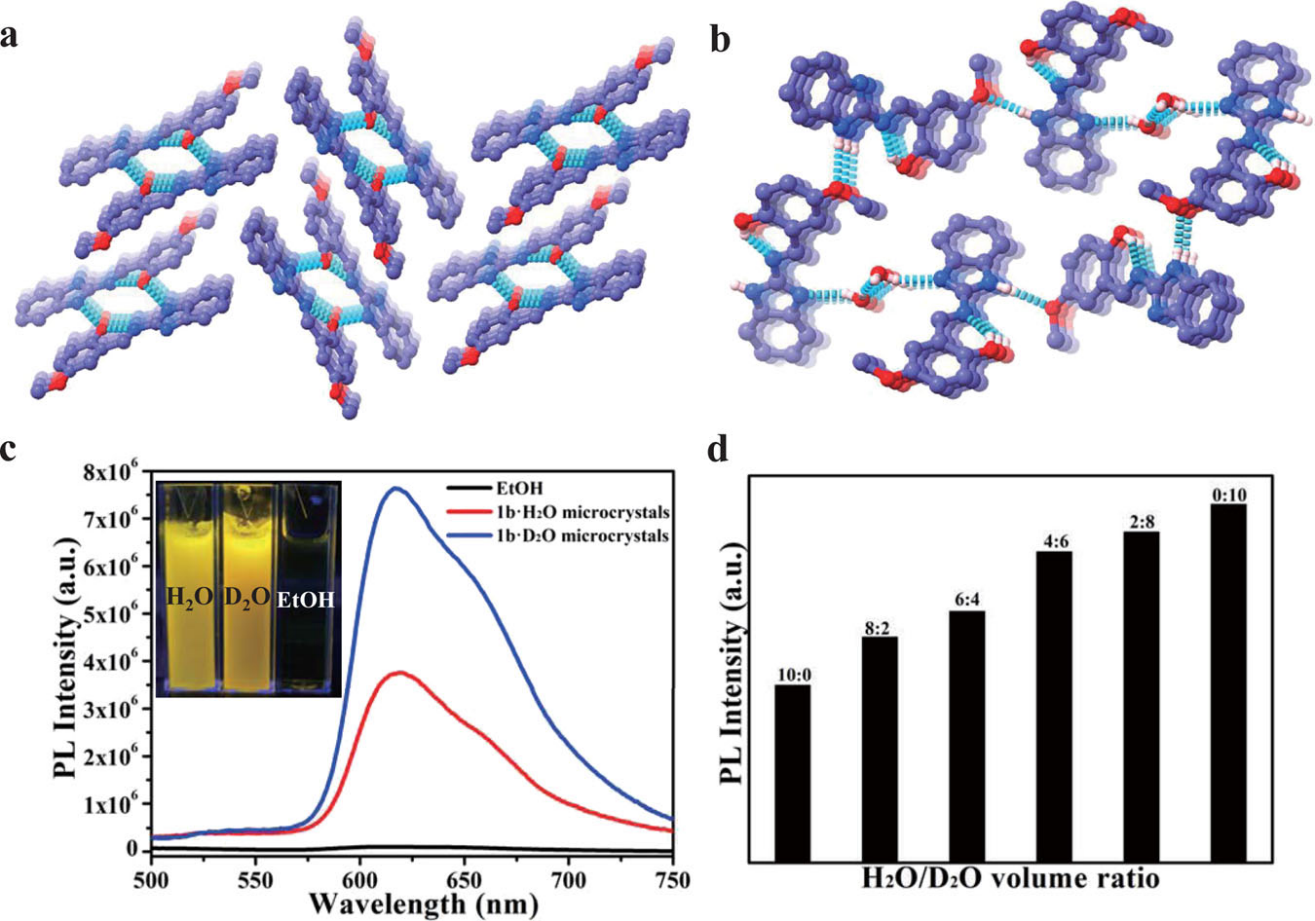
Molecular stacking and photoluminescence results. Molecular stacking of prepared single crystals: **a** **1b** and **b** **1b·H**_**2**_**O**. Multiple intermolecular and intramolecular hydrogen bonds in the single crystals are denoted by dotted lines. **c** Emission spectra (*λ*_ex_ = 400 nm) of **1b** (1 × 10^−5^ M) in ethanol and **1b·H**_**2**_**O (D**_**2**_**O)** microcrystal dispersion in water or deuterium oxide; **d** changes in relative photoluminescence intensity with respect to different ratios of H_2_O and D_2_O.

Target compounds **1a**, **1b**, and **1c** were prepared in high yields by one-step aldimine condensations of commercially available materials (Supplementary Figs. 1, 5, and 9). These compounds were characterized by ^1^H and ^13^C nuclear magnetic resonance (NMR) spectroscopies and mass spectrometry (Supplementary Figs. 2–4, 6–8, and 10–12). A methoxy group was introduced at different substitution positions in compounds **1b** and **1c** to investigate the effect of electron-donating groups on the optical properties of the materials. The presence of electronegative nitrogen and oxygen atoms, and active hydrogen atoms (N–H and O–H) promoted the formation of intramolecular and intermolecular hydrogen bonding (Supplementary Tables 7 and 8) between the organic molecules and with polar guest molecules, such as water (Fig. 2b, Supplementary Figs. 17, 19, and 20).

### Single crystals of target compounds

The single-crystal structures of the small organic compounds showed that all structures possessed intramolecular and intermolecular hydrogen bonds. Molecules of **1a** interlinked to form a one-dimensional (1D) supramolecular chain (Supplementary Fig. 17). Two molecules of **1b** interlinked to form a dimer via two N–H···O hydrogen bonds, with these dimers forming a 1D supramolecular chain (Fig. 2a). Four molecules of **1c** packed together to form a supramolecular ring via N–H···O and N–H···N intermolecular hydrogen bonds (Supplementary Fig. 20). The molecular configuration of **1a** single crystals showed a dihedral angle between R_1_ (benzimidazole ring) and R_2_ (phenyl ring) of about 18.56° (Supplementary Fig. 18). An intramolecular hydrogen bond between the hydrogen atom of a hydroxyl group and the nitrogen atom of a C=N bond (N∙∙∙H distance, 1.892 Å) and one intermolecular hydrogen bond between two adjacent molecules (N–H∙∙∙N distance, 2.133 Å or 2.072 Å) supported the aforementioned organization of the **1a** molecules (Supplementary Fig. 17).

### Single crystals of target compounds with H_2_O or D_2_O

In organic crystal growth, the presence of H_2_O or D_2_O molecules induces reorganization of the supramolecular array of organic compounds. Although the supramolecular structures of the three compounds were distinctive, the water-containing single-crystal structures of **1a** and **1c** were similar. The stoichiometry of water molecules in the unit cell of hydrated single crystals was 1:1. The unit cell dimensions of hydrated single crystals of **1a** and **1c** were larger than those of their anhydrous counterparts. For compound **1b**, the unit cell dimensions of hydrated single crystals were smaller than those of their anhydrous counterparts (Supplementary Tables 1–3). Taking compound **1a** as an example, a water molecule acted as a tridentate H-bonding moiety to link three surrounding molecules into a 1D ladder-shaped superstructure (Supplementary Fig. 17). The dihedral angles between aromatic segments R1 and R2 in the **1a·H**_**2**_**O** and **1a·D**_**2**_**O** lattices were about 10.98° and 10.99°, respectively (Supplementary Fig. 18), indicating that molecule **1a** in the **1a·H**_**2**_**O** and **1a·D**_**2**_**O** lattices was closer to planar compared with the anhydrous form. Molecules of **1a** formed a layer-by-layer structure indicative of face-to-face stacking. In each layer, molecular packing in a shifted cofacial arrangement was promoted. The molecules were stacked on top of each other and into columns along the *b* axis. Multiple intermolecular bonds between the organic molecules and H_2_O or D_2_O were beneficial for molecular planarity and acted as the driving force for formation of the layer-by-layer structure in the single-crystal state.

### Characterization of microcrystals

Well-defined microcrystals (**1a·H**_**2**_**O (D**_**2**_**O)**, **1b·H**_**2**_**O (D**_**2**_**O)**, and **1c·H**_**2**_**O (D**_**2**_**O)**) were fabricated using a facile self-assembly method (see Supplementary Information). The crystallisation process was accelerated by adding H_2_O or D_2_O to molecular solutions in ethanol. Scanning electron microscopy (SEM) images of the as-prepared crystalline samples showed a large number of microcrystals (Supplementary Figs. 21–23). X-ray diffraction (XRD) analysis in reflection mode was performed to characterize the molecular organization in different states. XRD patterns of the bulk polycrystalline powders and hydrated microcrystals were in agreement with the simulated patterns from the corresponding single crystals (Supplementary Fig. 24) and hydrated single crystals (Supplementary Fig. 25). For example, microcrystals of **1a·H**_**2**_**O** and **1a·D**_**2**_**O** had similar XRD patterns (Supplementary Fig. 24a). In contrast to the XRD pattern of **1b·H**_**2**_**O (D**_**2**_**O)** single crystals, **1a·H**_**2**_**O (D**_**2**_**O)** microcrystals had only three Bragg reflection peaks, suggesting that **1a·H**_**2**_**O (D**_**2**_**O)** microcrystals had a preferential growth orientation. Therefore, molecules in **1a·H**_**2**_**O** (D_2_O) microcrystals assembled preferentially along the (100), (002), and (004) lattice directions with good crystallinity.

### Optical properties

Hydrated single crystals and microcrystals of **1a**, **1b**, and **1c** showed significantly enhanced fluorescence compared with their solutions and anhydrous powders (Fig. 2c, Supplementary Figs. 28 and 29). We investigated the photophysical properties of all synthesized compounds in different states, namely, in solution, as anhydrous crystals, and as hydrated crystals. Supplementary Figs. 26a–26c show UV-visible absorption spectra of the chromophores in different anhydrous organic solvents (methanol, ethanol, tetrahydrofuran, and dichloromethane). The absorptions of the three compounds in the region of 320–450 nm was assigned to π–π* excitation of the enol ground state (E) to the first enol excited state (E*) (S_0_ → S_1_). We also investigated the PL properties of these compounds in different organic solvents (Supplementary Fig. 27). The formation of the keto excited state (K*) occurred through the excited-state intramolecular proton transfer (ESIPT) process following photoexcitation (E → E*) of the enol ground states, as shown in Supplementary Fig. 33a. All chromophores showed almost negligible fluorescence with dual emission peaks (Supplementary Table 4) upon excitation. The position of the methoxy group significantly influenced the proton transfer event in the excited state. The short-wavelength emission of the compounds at 459 nm was attributed to the excited state enol form (E*) (enol emission), while peaks at 594 nm (**1a**), 620 nm (**1b**), and 572 nm (**1c**) were attributed to the excited state keto form (keto emission) through a four-level photocycle (Supplementary Fig. 33a). Methoxy group substitution at different positions had a negligible impact on enol emission wavelength, but significantly affected the keto emission wavelengths. In the K* state, the methoxy group at the 5-position in **1b** further increased the electron density of the highest occupied molecular orbital (HOMO), which decreased the energy gap of the keto tautomer. Therefore, the ESIPT emission peak of **1b** was red-shifted compared with that of **1a**. However, when the methoxy group was located at the 4-position in **1c**, the conjugation effect was hindered and an inductive effect occurred. This increased the energy level of the lowest unoccupied molecular orbital (LUMO) and increased the energy gap of the keto tautomer. Therefore, the ESIPT emission of **1c** was blue-shifted compared with that of **1a**. These molecules were all typical ESIPT chromophores. C=N isomerization is the predominant nonradiative decay process of excited states in compounds with an unbridged C=N structure, such that solutions of the three compounds were nonfluorescent^35–37^.

Compared with UV-visible absorption spectra of solutions, the absorption spectra of microcrystals dispersed in aqueous solution were blue-shifted due to the formation of hydrated microcrystals (Supplementary Figs. 26d–26f). This blue-shift in the absorption spectra of hydrated microcrystals indicated the formation of H-aggregation, which was caused by cofacial stacking of the molecules. Subsequently, the emission spectra of hydrated microcrystals dispersed in water were investigated. A dramatic increase in the fluorescence intensity of hydrated microcrystals (microcrystals of **1a·H**_**2**_**O (D**_**2**_**O)**, **1b·H**_**2**_**O (D**_**2**_**O)**, and **1c·H**_**2**_**O (D**_**2**_**O)**) was observed. Water (and D_2_O) greatly increased the fluorescence quantum yield (QY) of these microcrystals (Supplementary Table 5), and the emission wavelengths of the six microcrystals were consistent with the respective molecular keto emissions in solution. The emission observed for the enol form in solution was absent in the microcrystals. The QY (*Φ*_*f*_ = 15.3%) of **1a·H**_**2**_**O** microcrystals was about 55 times higher than that in ethanol solution (*Φ*_*f*_ = 0.28%) and other organic solvents (Supplementary Table 5), accompanied with a red-shift to 593 nm. Steady-state fluorescence emission spectra of the hydrated single crystals (Supplementary Fig. 28) showed similar emission behaviour to the corresponding microcrystals. Steady-state fluorescence emission spectra of the molecular polycrystalline powders were also investigated (Supplementary Fig. 28). All compounds were weakly emissive with fairly low fluorescence QYs. The emission wavelengths of polycrystalline powders of **1a**, **1b**, and **1c** were blue-shifted compared with their individual hydrated single crystals and microcrystals.

**Fig. 3:**
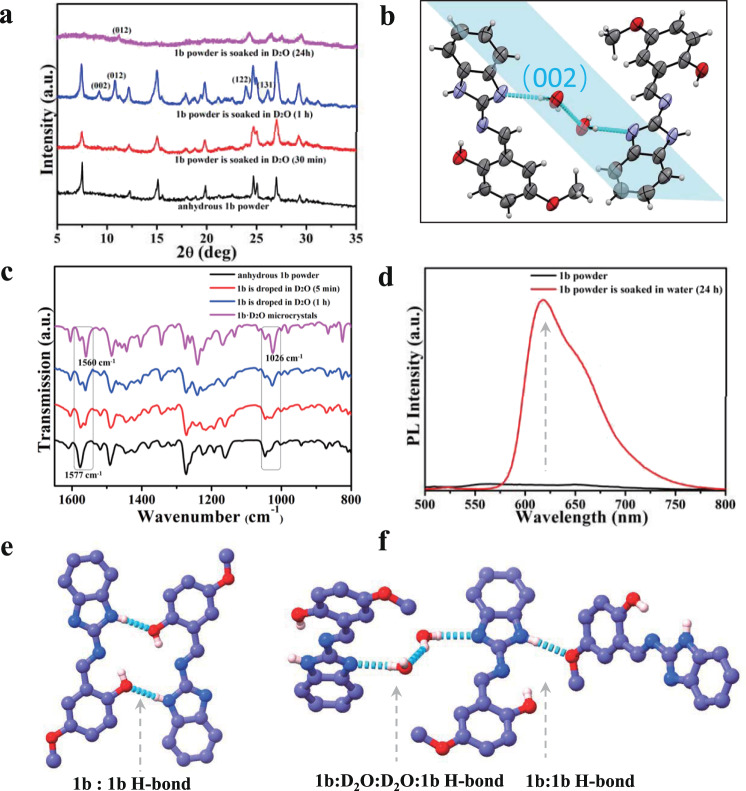
Characterization of 1b and 1b·D_2_O. In situ XRD (**a**), FT-IR (**c**) and emission (**d**) spectra of **1b** polycrystalline powder soaked in D_2_O. **b** Molecular arrangement in the (002) lattice direction of **1b·D**_**2**_**O** single crystals; **e** N–H···O (–OD) hydrogen bond between molecules in the anhydrous structure; **f** N–H···O (–OCH_3_) hydrogen bond between molecules, N···D (D_2_O) hydrogen bond between molecules and water.

As **1b** showed the fastest water-binding rate among the three molecules, **1b** was used to explore the in situ formation of new hydrogen bonds with guest water molecules. To avoid the influence of the OH-stretching vibration in H_2_O, D_2_O was used for in situ XRD and Fourier transform infrared (FT-IR) spectroscopy analyses. When the polycrystalline powder of anhydrous **1b** was dipped in D_2_O, in situ XRD measurements showed new Bragg reflection peaks assigned to **1b·D**_**2**_**O** (Fig. 3a). For example, (002), (012), (122), and (131) lattice directions appeared when **1b** polycrystalline powder was dipped in D_2_O for 1 h. When the sample was dipped for almost 24 h, the XRD pattern matched well with that of the aforementioned **1b·D**_**2**_**O** microcrystals, indicating successful in situ transformation from the anhydrous state to the hydrated state. Interestingly, new peaks for the (002) lattice direction occurred when anhydrous **1a** and **1b** polycrystalline powders were dipped in D_2_O. The (002) lattice direction is the orientation plane in which water and organic molecules form new intermolecular hydrogen bonds in the anhydrous crystal lattices. In situ FT-IR spectra (Fig. 3c) of **1b** polycrystalline powder with D_2_O showed a stretching band at 1577 cm^−1^ that diminished gradually and a new stretching band at 1560 cm^−1^ that appeared simultaneously. This change suggested that the N–H chemical environment in the benzimidazole ring changed after being dipped in D_2_O. Combined with single-crystal data analysis, the disappearance of the stretching band at 1577 cm^−1^ was attributed to breakage of the N–H···O (–OH) hydrogen bond between organic molecules in the anhydrous structure (Fig. 3e), while the new stretching band at 1560 cm^−1^ suggested the formation of N–H···O (–OCH_3_) hydrogen bonds between molecules in the hydrated structure (Fig. 3c). Another new stretching band at 1025 cm^−1^ was attributed to the formation of a N···D (D_2_O) hydrogen bond (Fig. 3f). The location of the methoxy group of **1b** in Fig. 3e and f has changed due to the formation new hydrogen bonds between **1b** molecules.

### Possible mechanism

**Fig. 4:**
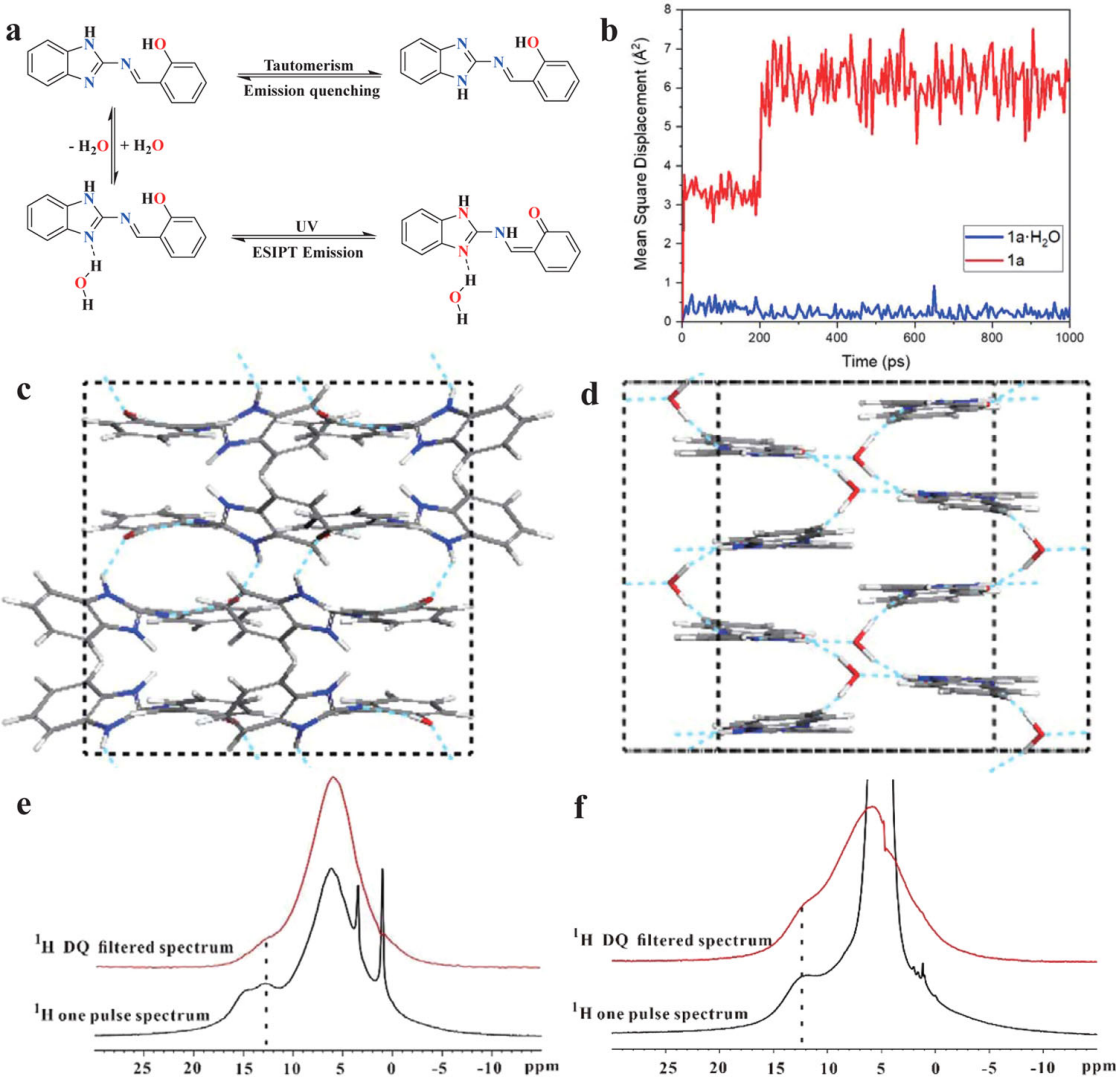
Proposed activation of emission mechanism. **a** Proposed activation of single-molecule emission mechanism for **1a·H**_**2**_**O**; **b** mean square displacement of **1a** and **1a·H**_**2**_**O** microcrystals during 1-ns molecular dynamics simulation; optimised molecular crystal structures of **c** **1a** and **d** **1a·H**_**2**_**O** single crystals at the final step (sky-blue dotted lines represent the hydrogen bond network); solid-state ^1^H NMR spectra of **e** **1a** and **f** **1a·H**_**2**_**O**. ^1^H double quantum filtered (DQ-filtered) spectra (in red) were recorded for comparison with ^1^H one-pulse spectra (in black), where signals of protons with high local mobility were filtered.

To understand the fluorescence turn-on properties of the hydrated crystals, theoretical calculations were conducted. The emission wavelength of hydrated crystals (**1a·H**_**2**_**O (D**_**2**_**O)**, **1b·H**_**2**_**O (D**_**2**_**O)**, and **1c·H**_**2**_**O (D**_**2**_**O)**) was similar to that of the keto emission in an anhydrous organic solvent, while the enol emission was not observed. This showed that intramolecular hydrogen bonding was more effective in the solid state with the ESIPT process. The active hydrogen atom in the benzimidazole ring was responsible for tautomer formation and shuttled between its two N atoms (Fig. 4a), resulting in fluorescence quenching. Using the polycrystalline powder of **1a**, the radiation rate (*kr* ~ *E*^*2*^*f*) in the powder state was very low owing to the low *f* value (*f* = 0.0123). Therefore, the fluorescence quantum efficiency of ESIPT emissions (K*) from polycrystalline powders of **1a**, **1b**, and **1c** was low owing to the nonradiative deactivation pathways (nπ*) of the tautomer (Supplementary Table 7 and Supplementary Fig. 33b). Water acting as a hydrogen bond donor formed strong intermolecular hydrogen bonds (N∙∙∙H–O or N∙∙∙D–O) with the nitrogen atom of the molecule (Fig. 4a), inhibiting tautomer formation. When the tautomer was inhibited by intermolecular hydrogen bond formation in the excited states, the electronic transition of geometry-B (S1) (Supplementary Fig. 34) changed from nπ* to lower-energy ππ*, resulting in enhanced keto (K*) emission in the solid state. The radiation rate (*kr* ~ *E*^*2*^*f*) of **1a·H**_**2**_**O** was higher than that of **1a** polycrystalline powder owing to the higher *f* value (*f* = 0.1934) (Supplementary Table 9 and Supplementary Fig. 34). Therefore, hydrated crystals **1a·H**_**2**_**O (D**_**2**_**O)**, **1b·H**_**2**_**O (D**_**2**_**O)**, and **1c·H**_**2**_**O (D**_**2**_**O)** exhibited relatively bright fluorescence in the solid state. Compared with water, deuterium oxide showed a stronger proton donating ability toward the N atom of the benzimidazole group. Therefore, the deuterium isotope effect caused the fluorescence quantum yields of D_2_O-containing hydrates to be higher than those of H_2_O-containing hydrates (Supplementary Table 5)^38^. The fluorescent images also showed fluorescence intensity of **1a·D**_**2**_**O** microcrystals grown on silicon dioxide substrate was stronger than that of **1a·H**_**2**_**O** microcrystals grown on silicon dioxide substrate (Supplementary Fig. 39).

A 1-ns molecular dynamics simulation was also conducted using the COMPASS force field to investigate the molecular stacking mode and hydrogen-bonding network. Quantitative mean square displacements of dynamic trajectories, as shown in Fig. 4b, indicated a larger thermal fluctuation in the **1a** molecular crystal than in **1a·H**_**2**_**O**. The corresponding equilibrium structures are also shown in Fig. 4c and d, respectively. This verified that a supramolecular ring was present via N–H···O and N–H···N hydrogen bonding in the single crystal of **1a**. This interaction induced a twist between R1 and R2, but also formed a dimer structure between the organic molecules that exhibited certain flexibility with high freedom. However, for the single crystal of **1a·H**_**2**_**O**, water acted as a “glue” to connect the organic molecules, with a well-organized three-dimensional hydrogen network lowering the fluctuation displacement and protecting the planarity of the organic molecule. Therefore, aided by water, the hydrogen-bonding network in **1a·H**_**2**_**O** single crystals was prone to forming a relatively confined space to suppress the vibration of **1a** molecules, which effectively decreased loss from electronic transitions between orbitals and further enhanced molecular photoluminescence.

To further confirm the existence of hydrogen bonds in **1a·H**_**2**_**O**, solid-state 1D ^1^H NMR double quantum filtered (DQ-filtered) spectra and 1H one-pulse spectra were recorded. Figure 4e shows the ^1^H one-pulse spectrum of **1b**. The resonances at 12.8 and 14.8 ppm were associated with the –OH and –NH units, respectively. However, these two signals were not observed in the ^1^H DQ-filtered spectrum (Fig. 4f), indicating that the –OH and –NH protons of **1a** exhibited high local mobility. In contrast to **1a**, **1a·H**_**2**_**O** showed only one peak at 12.0 ppm in the ^1^H one-pulse spectrum. This was in agreement with the single crystal structure, in which hydrogen bonding with water induced identical local structures for the –OH and –NH protons. The signal at 12.0 ppm was also observed in the ^1^H DQ-filtered spectrum, implying that –OH and –NH protons in **1a·H**_**2**_**O** had limited local mobility, which might be induced by hydrogen bonding with water. To further verify the mechanism, compound (2-(((1-methyl-1H-benzo[*d*]imidazol-2-yl)imino)methyl)phenol (named as **1d**)) (Supplementary Fig. 32) was prepared, in which the active hydrogen atom on the N atom present in **1b** was replaced by a methyl group (Supplementary Fig. 13). Compound **1d** strongly fluoresced in the solid state owing to the lack of tautomer formation after replacing the active hydrogen in the benzimidazole moiety.

The hydrated microcrystals were also found to be metastable structures. Using **1a·H**_**2**_**O** microcrystals as an example, the XRD pattern changed significantly after heat treatment, becoming similar to that of bulk **1a** polycrystalline powder, indicating a rearrangement of molecules in the crystal lattice (Supplementary Fig. 37). Furthermore, the surfaces of the hydrated microcrystals were covered with small dots after thermal treatment at 60 °C for 25 min, which might have been caused by water loss (Supplementary Figs. 21–23). Upon thermal treatment, a **1a·H**_**2**_**O** microcrystal film deposited on a polytetrafluoroethylene (PTFE) membrane (Supplementary Fig. 34) showed gradually decreasing fluorescence with prolonged heating. The fluorescence quenching of the **1a·H**_**2**_**O** film deposited on a PTFE membrane after heat treatment was turned on by adding water. Other microcrystals have shown similar fluorescence on-off switching behaviour. Owing to the large gap in quantum yield between **1b·H**_**2**_**O** microcrystals and **1b·D**_**2**_**O** microcrystals (Supplementary Table 5), **1b** was used as a fluorescent probe to investigate different ratios of H_2_O and D_2_O (Fig. 2d).

## Discussion

Organic hydrate luminogens were prepared and their water-promoted fluorescence enhancement properties were investigated. Intercalated water molecules played an important role in triggering molecular reorganization in the hydrated crystalline state by forming new intermolecular hydrogen bonds between the organic molecules and guest water molecules. This water-induced rearrangement of the organic compounds in the crystalline state caused significant enhancement of their molecular emissions in the solid state by suppressing shuttling of the active hydrogen atom between N atoms in the benzimidazole ring. Water molecules acting as hydrogen bond donors not only changed the molecular stacking mode in the hydrated crystals, but also markedly altered the electronic transition of the molecular keto form from nπ* to ππ* through the ESIPT process. This process enabled these small organic compounds to optically sense heavy water and quantify the ratio of H_2_O to D_2_O. This study provides a basis for the design and development of functional organic hydrate luminogens that exhibit water-induced fluorescence enhancement for solid-state device applications.

## Methods

### Synthesis of 2-(((1H-benzo[*d*]imidazol-2-yl)imino)methyl)-4-methoxyphenol (1a)

A mixture of 2-aminobenzimidazole (1.33 g, 10 mmol), salicylaldehyde (1.22 g, 10 mmol) and formic acid (0.1 mL) in ethanol (60 mL) was refluxed with vigorous stirring for 6 h. The precipitated compound was filtered and washed with ethanol (yield: 86 %). The product was confirmed by ^1^H NMR, ^13^C NMR, HR-MS. ^1^H NMR (400 MHz, DMSO-*d*_*6*_) δ (ppm): 12.81 (s, 1H), 12.15 (s, 1H), 9.69 (s, 1H), 7.89 (d, *J* = 6.9 Hz, 1H), 7.61 (s, 1H), 7.51 (dd, *J* = 11.2, 4.1 Hz, 2H), 7.22 (dd, *J* = 5.9, 3.1 Hz, 2H), 7.04 (t, *J* = 8.4 Hz, 2H). ^13^C NMR (151 MHz, DMSO-*d*_*6*_) δ (ppm) 166.03, 161.03, 154.34, 135.06, 132.89, 122.59, 120.06, 119.82, 117.31. HRMS (TOF MS EI^+^) calculated for C_14_H_11_N_3_O 237.0902, found 237.0905.

### Synthesis of 2-(((1H-benzo[*d*]imidazol-2-yl)imino)methyl)phenol (1b)

A mixture of 2-aminobenzimidazole (1.33 g, 10 mmol), 2-hydroxy-5-methoxybenzaldehyde (1.52 g, 10 mmol) and formic acid (0.01 mL) in ethanol (80 mL) was refluxed with vigorous stirring for 6 h. The precipitated compound was filtered and washed with ethanol (yield: 91%). The product was confirmed by ^1^H NMR, ^13^C NMR, HRMS (TOF MS EI^+^). ^1^H NMR (400 MHz, DMSO-*d*_*6*_) δ 12.77 (s, 1H), 11.47 (s, 1H), 9.67 (s, 1H), 7.60 (s, 1H), 7.47 (d, *J* = 3.0 Hz, 2H), 7.20 (dd, *J* = 5.9, 3.1 Hz, 2H), 7.12 (dd, *J* = 9.0, 3.1 Hz, 1H), 6.97 (d, *J* = 9.0 Hz, 1H), 3.77 (s, 3H). ^13^C NMR (151 MHz, DMSO-*d*_*6*_) δ (ppm) 165.17, 155.22, 154.72, 152.62, 142.83, 122.73, 122.38, 119.90, 119.05, 118.33, 114.18, 111.66, 55.98. HRMS (TOF MS EI^+^) calculated for C_15_H_13_N_3_O_2_ 267.1008, found 267.1005.

### Synthesis of 2-(((1H-benzo[*d*]imidazol-2-yl)imino)methyl)-5-methoxyphenol (1c)

A mixture of 2-aminobenzimidazole (1.33 g, 10 mmol), 2-hydroxy-4-methoxybenzaldehyde (1.52 g, 10 mmol) and formic acid (0.01 mL) in ethanol (50 mL) was refluxed with vigorous stirring for 6 h. The precipitated compound was filtered and washed with ethanol (yield: 83%). The product was confirmed by ^1^H NMR, ^13^C NMR, and HRMS (TOF MS EI^+^). ^1^H NMR (400 MHz, DMSO-*d*_*6*_) δ 12.71 (s, 1H), 12.64 (s, 1H), 9.54 (s, 1H), 7.83–7.73 (m, 1H), 7.57 (s, 1H), 7.44 (s, 1H), 7.18 (d, *J* = 3.6 Hz, 2H), 6.69-6.55 (m, 2H), 3.84 (s, 3H). ^13^C NMR (151 MHz, DMSO-*d*_*6*_) δ (ppm) 165.59, 165.26, 163.60, 154.45, 142.86, 135.10, 134.38, 122.44, 122.23, 118.78, 113.35, 111.51, 108.17, 101.34, 56.10. HRMS (TOF MS EI^+^) calculated for C_15_H_13_N_3_O_2_ 267.1008, found 267.1006.

### Synthesis of 2-(((1-methyl-1H-benzo[*d*]imidazol-2-yl)imino)methyl)phenol (1d)

A mixture of 2-amino-1-methylbenzimidazole (1.47 g, 10 mmol), salicylaldehyde (1.22 g, 10 mmol) and formic acid (0.01 mL) in ethanol (100 mL) was refluxed with vigorous stirring for 6 h. The precipitated compound was filtered and washed with ethanol (yield: 87%). The product was confirmed by ^1^H NMR, ^13^C NMR, and HRMS (TOF MS EI^+^). ^1^H NMR (400 MHz, DMSO-*d*_*6*_) 11.67 (s, 1H), 9.72 (s, 1H), 8.00 (d, *J* = 7.6 Hz, 1H), 7.59 (dd, *J* = 16.7, 7.2 Hz, 2H), 7.51 (t, *J* = 7.7 Hz, 1H), 7.33–7.16 (m, 2H), 7.03 (t, *J* = 8.7 Hz, 2H), 3.87 (s, 3H). ^13^C NMR (151 MHz, DMSO-*d*_6_) δ (ppm) 165.00, 160.84, 154.33, 141.64, 135.95, 135.40, 131.77, 122.81, 122.57, 120.49, 120.21, 119.14, 117.29, 110.72, 29.34. HRMS (TOF MS EI^+^) calculated for C_15_H_13_N_3_O 251.1059, found 251.1054.

### Preparation of 1a·H_2_O, 1a·D_2_O, 1b·H_2_O, 1b·D_2_O, 1c·H_2_O, and 1c·D_2_O microcrystals

In a typical procedure, a sample of **1a** in ethanol (500 μL, 1 × 10^−3^ M) was injected drop-wise into deionized H_2_O or D_2_O (99.8 %, 3 mL). Well-defined **1a·H**_**2**_**O** or **1a·D**_**2**_**O** microcrystals were prepared by a facile self-assembly method. Samples were left to stand for 6 h to allow stabilization.

## Supplementary information

Supplementary Information

## Data Availability

The authors declare that data supporting the findings of this study are available within the paper and its Supplementary Information, and also from the authors upon request. The X-ray crystallographic coordinates for structures reported in this study have been deposited at the Cambridge Crystallographic Data Centre (CCDC), under deposition numbers 1980949, 1847872, 1890749, 2055410, 2018980, 2018979, 2018982, 2018985, and 2018984. These data can be obtained free of charge from the Cambridge Crystallographic Data Centre via www.ccdc.cam.ac.uk/data_request/cif.
