# Supplementary material for: Unexpected organic hydrate luminogens in the solid state
Source: Nat Commun. 2021 Apr 20;12:2339. doi: 10.1038/s41467-021-22685-0 (PMC8058042; doi:10.1038/s41467-021-22685-0)
Supplement: Supplementary file 1 — Supplementary Information [file 41467_2021_22685_MOESM1_ESM.pdf]

Supplementary Information

Unexpected Organic Hydrate Luminogens in the Solid State

Feng Zhou,[1] Peiyang Gu,[1] Zhipu Luo,[2] Hari Krishna Bisoyi,[3] Yujin Ji,[4] Youyong Li,[4] Qingfeng Xu,[1] Quan Li,[3,5*] and Jianmei Lu[1*]

[1]*College of Chemistry, Chemical Engineering and Materials Science, Collaborative Innovation Center of Suzhou Nano Science and Technology, Soochow University, Suzhou, Jiangsu 215123, China. E-mail: lujm@suda.edu.cn*

[2]*Institute of Molecular Enzymology, School of Biology and Basic Medical Sciences, Soochow University, Suzhou, 215123, China.*

[3]*Advanced Materials and Liquid Crystal Institute and Chemical Physics Interdisciplinary Program, Kent State University, USA.*

[4]*Institute of Functional Nano & Soft Materials (FUNSOM), Jiangsu Key Laboratory for Carbon-Based Functional Materials & Devices, Soochow University, Suzhou, Jiangsu 215123, China.*

[5]*Institute of Advanced Materials and School of Chemistry and Chemical Engineering, Southeast University, Nanjing, Jiangsu Province 211189, China. E-mail: quanli3273@gmail.com*

Supplementary Eperimental details

Materials: 2-Aminobenzimidazole, deuterium oxide (99.8 atom % D), 2-hydroxy-4-methoxybenzaldehyde, 2-hydroxy-3-methoxybenzaldehyde, and 2-hydroxy-5-methoxybenzaldehyde were purchased from TCI. Formic acid and ethanol were purchased from Sinopharm Chemical Reagent Co., Ltd. (Shanghai, China). All reagents were used as received from commercial sources.

Apparatus: ^1H or ^{13}C NMR spectra were collected on an INOVA 400 MHz spectrometer with dimethyl sulfoxide ($CDCl_3$ or DMSO-d_6) as the solvent. UV-Vis spectra were measured by a Perkin-Elmer λ-17 spectrometer by using a 1 cm^2 quartz cell. Fluorescence spectra were recorded on an Edinburgh-920 fluorescence spectra photometer (Edinburgh Co., UK) with a slit width of 2 nm. The fluorescent quantum yield (QY) of the compounds in different solutions and microstructures was determined by using fluorescein (Φ_F = 79 % in 0.1 M NaOH) as the standard. The X-ray diffraction data were recorded on a Rigaku CCD X-ray diffractometer. X-ray powder diffraction (XRPD) for **1a, 1a·H_2O**, **1a·D_2O**, **1b, 1c·H_2O** and **1c·D_2O** was recorded on a Rigaku D/Max-2500 diffractometer at 40 kV and 100 Ma with a Cu-target tube and a graphite monochromator. X-ray powder diffraction (XRPD) for **1c** was recorded on a Bruker D8 VENTURE with a Ga-target tube. X-ray powder diffraction (XRPD) for **1b·H_2O** and **1b·D_2O** was determined by Microcrystal electron diffraction (MicroED). The sizes and shapes of the microstructures were observed by using a Hitachi S-4700 field-emission scanning electron microscope (SEM). Mass spectra were obtained using a GCT Premier high-resolution time-of-flight mass spectrometer and using EI as the ion source. The fluorescence lifetimes were measured on a Fluorolog-3 spectrometer. A 1-ns molecular dynamics simulation was conducted using the COMPASS force field to investigate the molecular stacking mode and hydrogen-bonding network.

Solid-state NMR(SSNMR) experimental condition: Solid-state NMR ^1H experiments were performed on a Bruker Avance III HD WB 400 spectrometer operating at a Larmor frequency of 400.25 MHz for ^1H equipped with a double-resonance magic-angle spinning (MAS) probe, supporting MAS rotors of 3.2

mm outer diameter. The rf nutation frequencies for ^1H was 78.1 kHz, corresponding to 3.2us for 90° pulse. All the experiments were conducted at MAS frequency of 15000 Hz. ^1H double quantum (DQ) filtered spectra were recorded using the back-to-back (BaBa) sequence.[1] All spectra were referenced with respect to tetramethylsilane (TMS) using solid adamantane (^1H, 1.85 ppm) as a secondary reference.[2]

X-ray diffraction crystallography: Single crystals of **1a**, **1b**, and **1c** were obtained by slow evaporation of the concentrated ethanol solution (10^{-1} mol/L) at room temperature. **1a·H₂O**, **1a·D₂O**, **1b·H₂O**, **1b·D₂O**, **1c·H₂O** and **1c·D₂O** single crystals were obtained by the slow evaporation of their solutions in ethanol/H_2O (15:1 v/v). The key parameters of the single crystal structures are listed in Supplementary Tables 1-3. CCDC1980949, 1847872, 1890749, 2055410, 2018980, 2018979, 2018982, 2018985, 2018984 contains the supplementary crystallographic data for this paper.

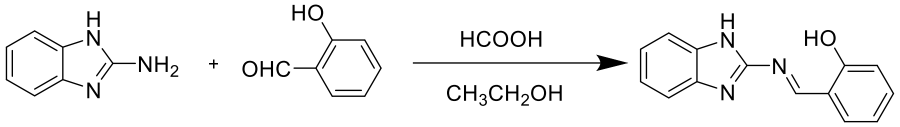

1a

Supplementary Figure 1. Synthesis and chemical structure of **1a**.

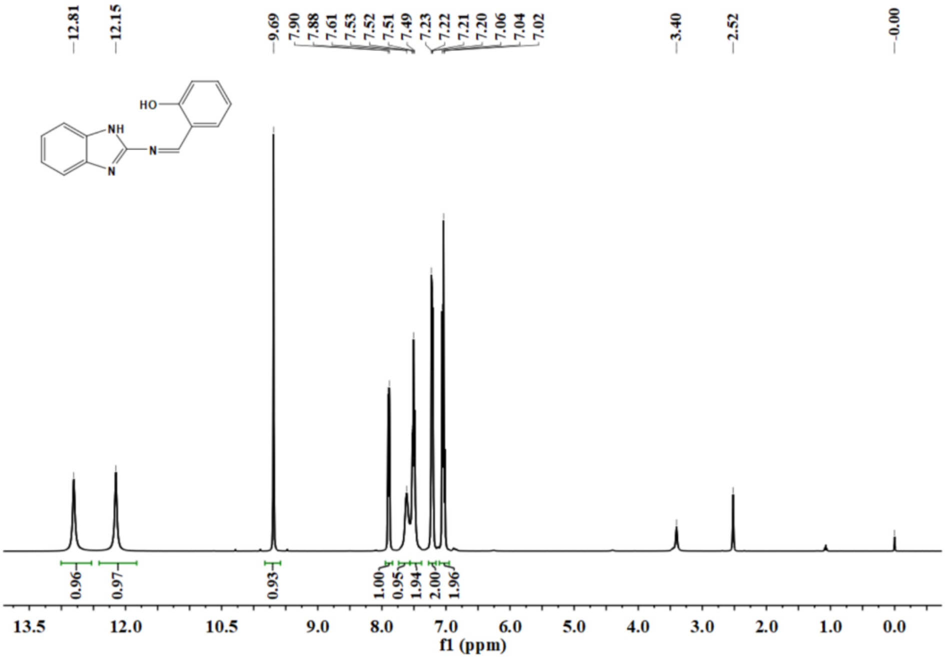

Supplementary Figure 2. ^1H NMR spectrum of **1a**.

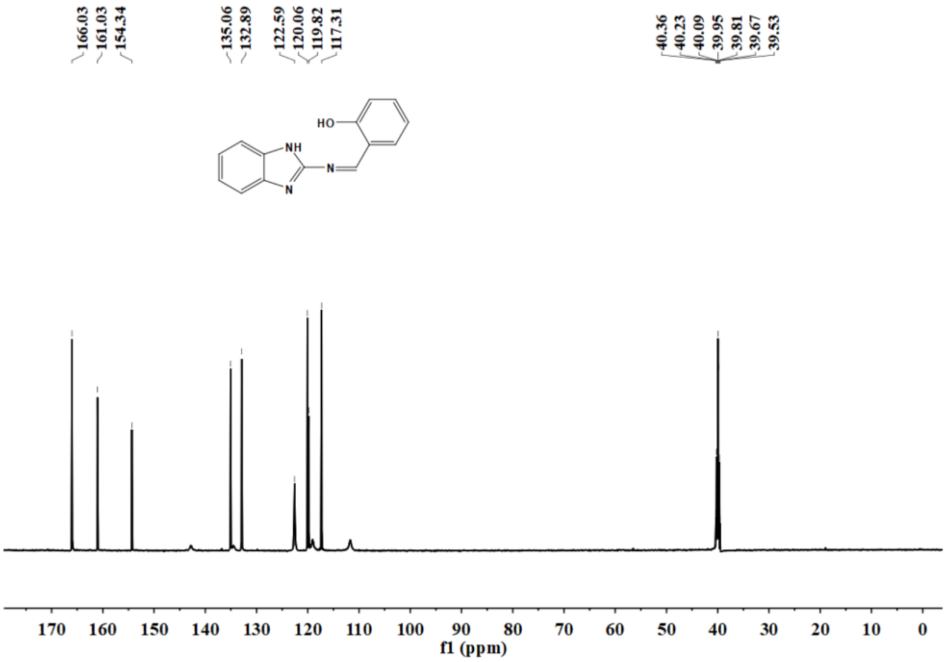

Supplementary Figure 3. ^{13}C NMR spectrum of **1a**.

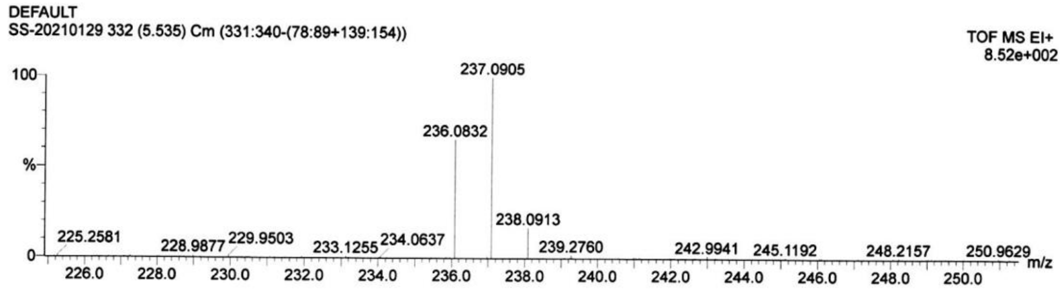

Minimum:	10.00				-1.5		
Maximum:	100.00		30.0	5.0	50.0		
Mass	RA	Calc. Mass	mDa	PPM	DBE	i-FIT	Formula
236.0832	65.20	236.0824	0.8	3.4	11.5	394.3	C14 H10 N3 16O
237.0905	100.00	237.0902	0.3	1.3	11.0	24.3	C14 H11 N3 16O

Supplementary Figure 4. The HRMS (TOF MS EI$^+$) spectrum of **1a**.

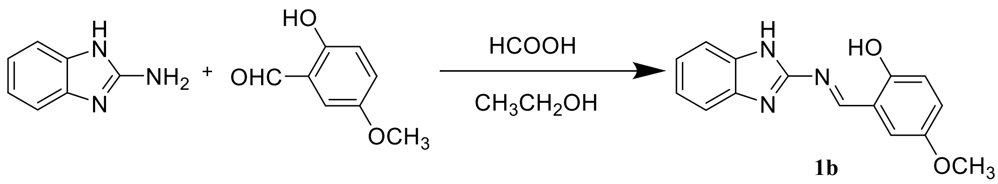

Supplementary Figure 5. Synthesis and chemical structure of **1b**.

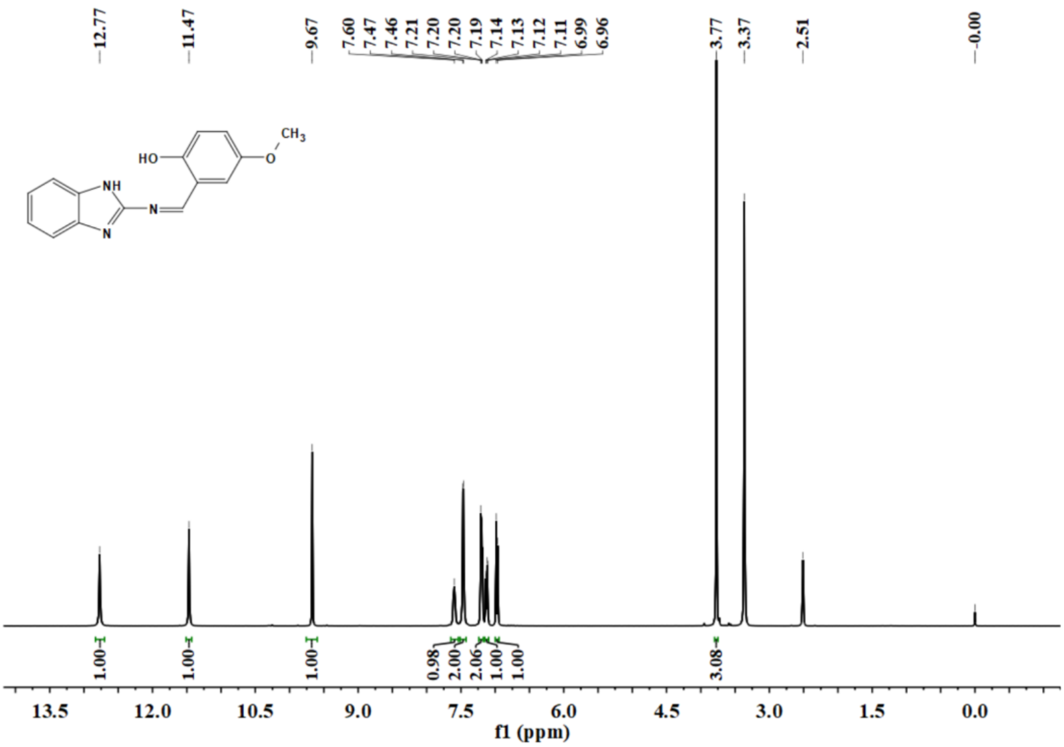

Supplementary Figure 6. ^{1}H NMR spectrum of **1b**.

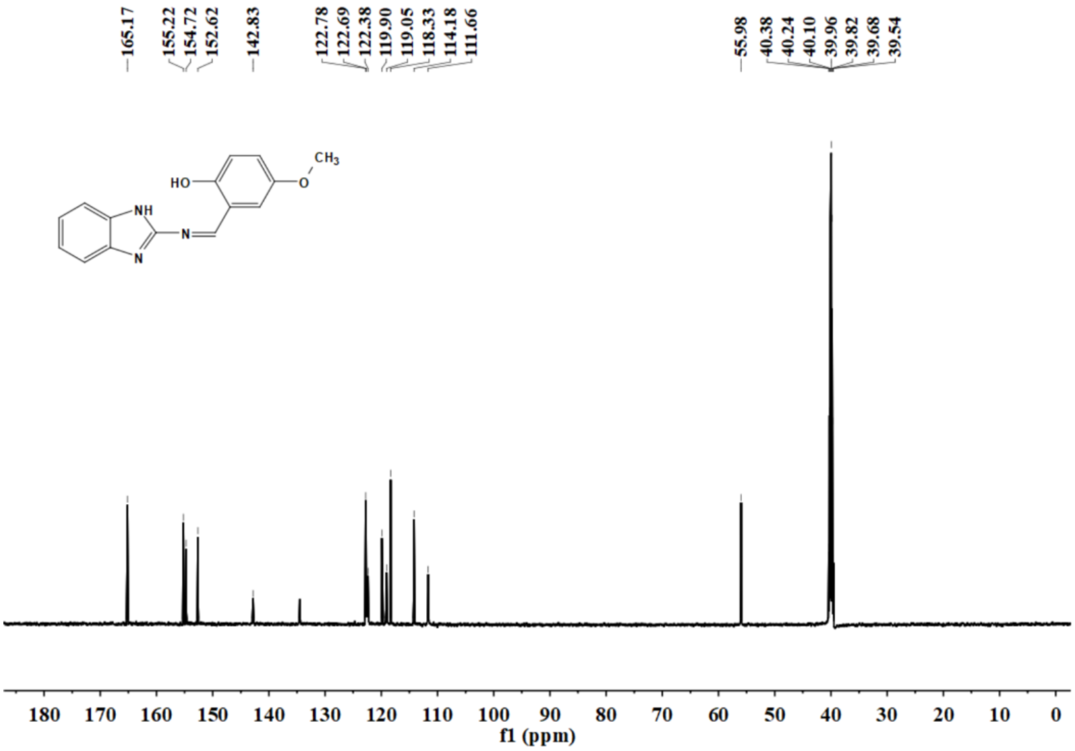

Supplementary Figure 7. ^{13}C NMR spectrum of **1b**.

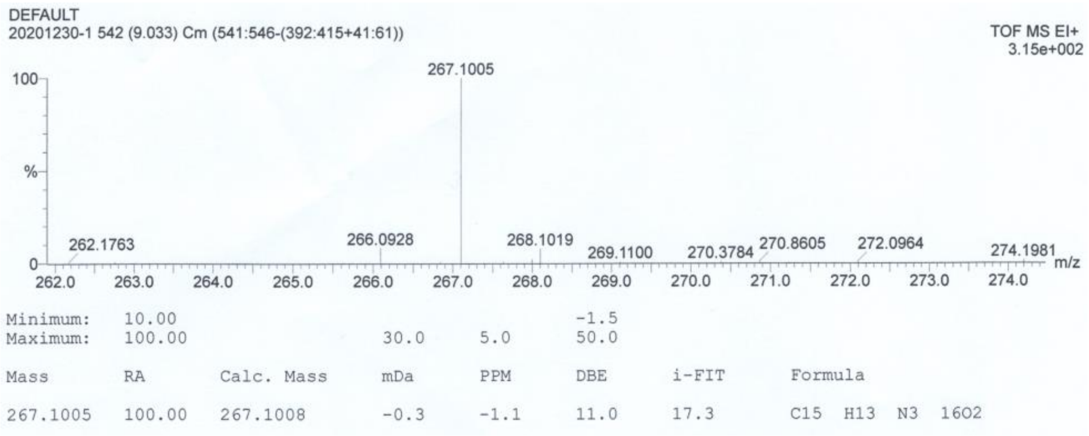

Supplementary Figure 8. The HRMS (TOF MS EI$^+$) spectrum of **1b**.

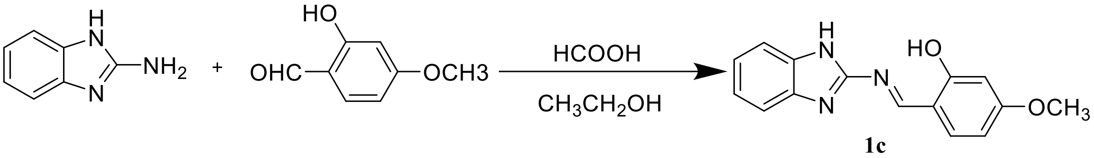

Supplementary Figure 9. Synthesis and chemical structure of **1c**.

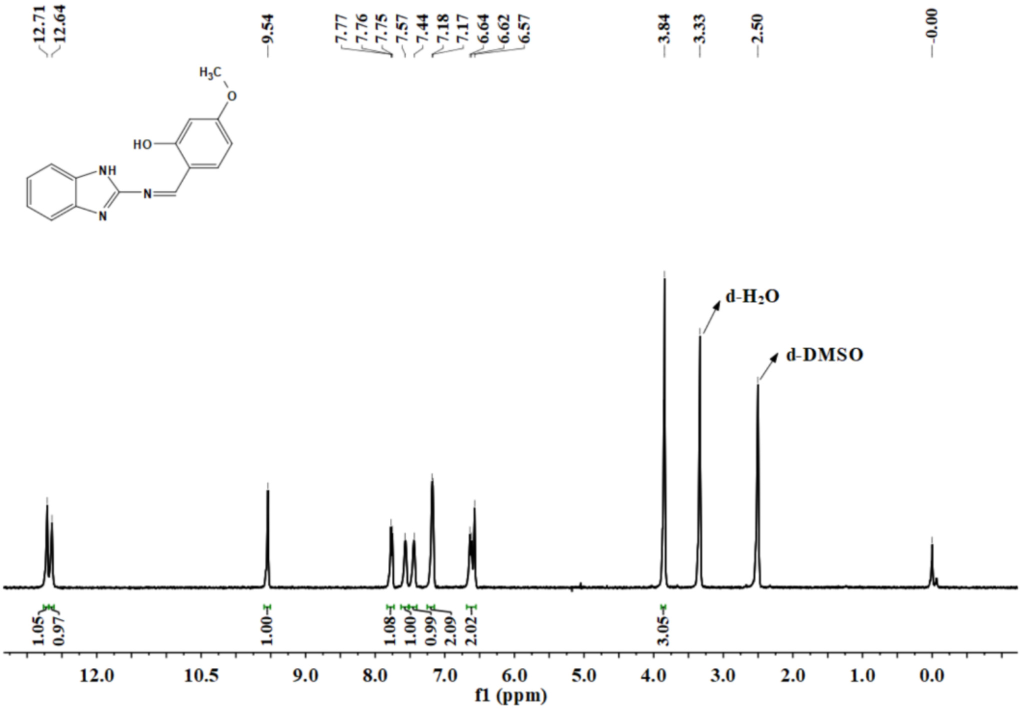

Supplementary Figure 10. ^{1}H NMR spectrum of **1c**.

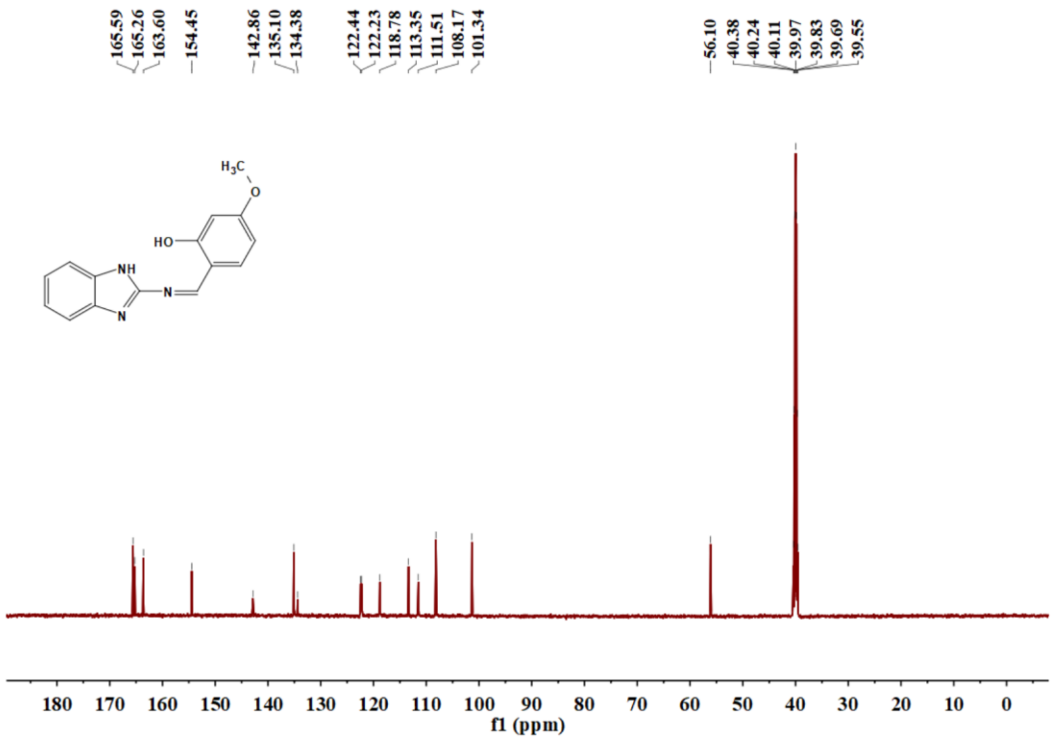

Supplementary Figure 11. ^{13}C NMR spectrum of **1c**.

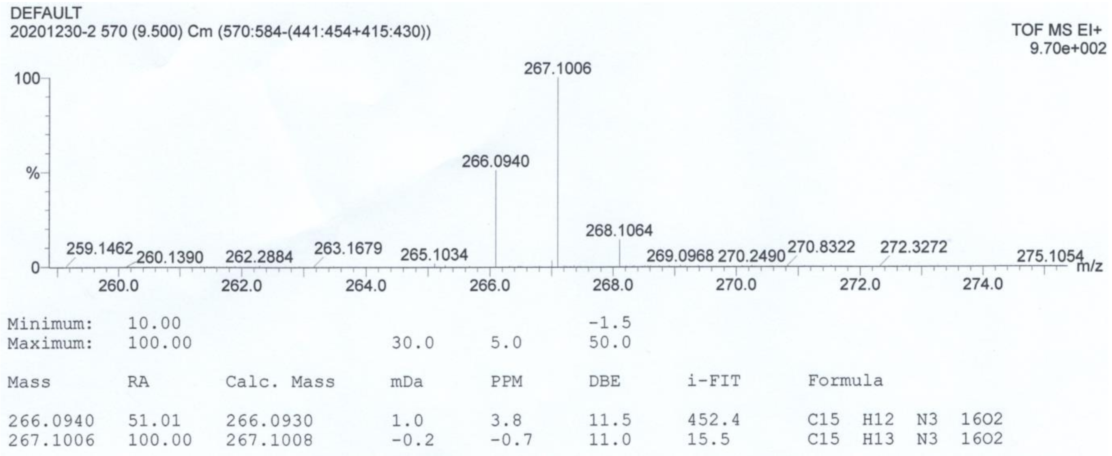

Supplementary Figure 12. The HRMS (TOF MS EI$^+$) spectrum of **1c**.

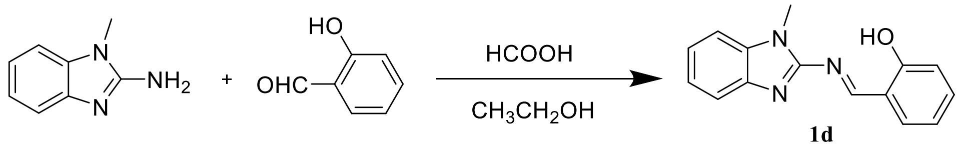

Supplementary Figure 13. Synthesis and chemical structure of **1d**.

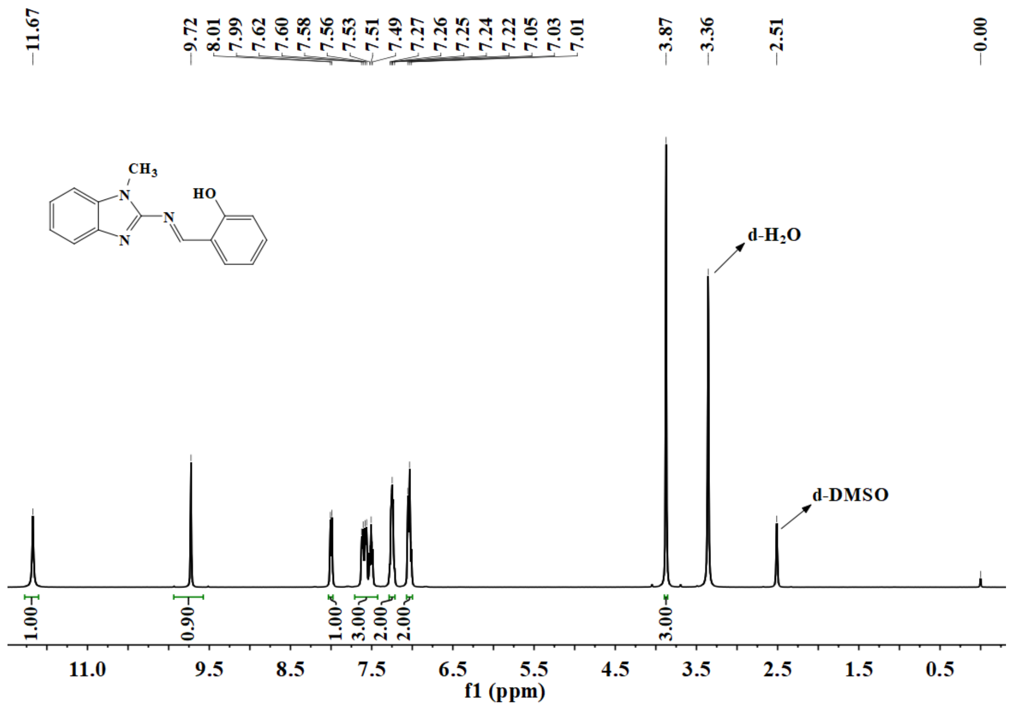

Supplementary Figure 14. ^{1}H NMR spectrum of **1d**.

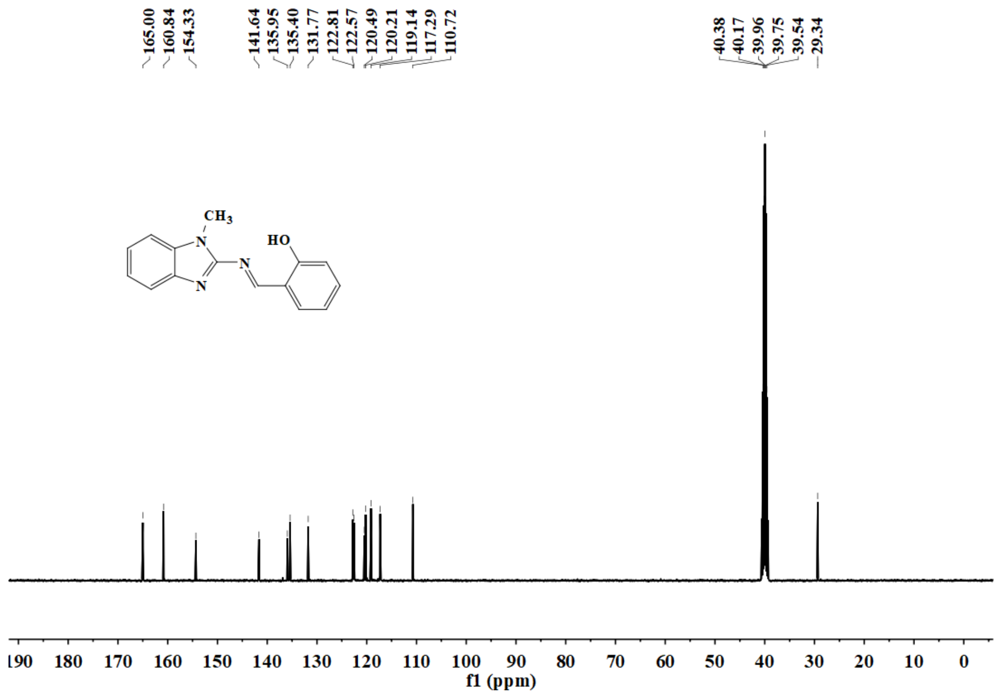

Supplementary Figure 15. ^{13}C NMR spectrum of **1d**.

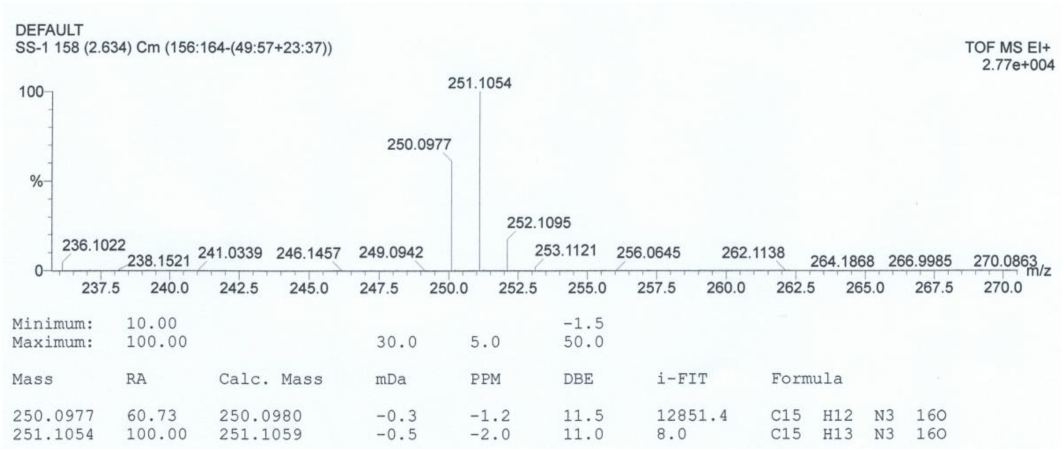

Supplementary Figure 16. The HRMS (TOF MS EI$^+$) spectrum of **1d**.

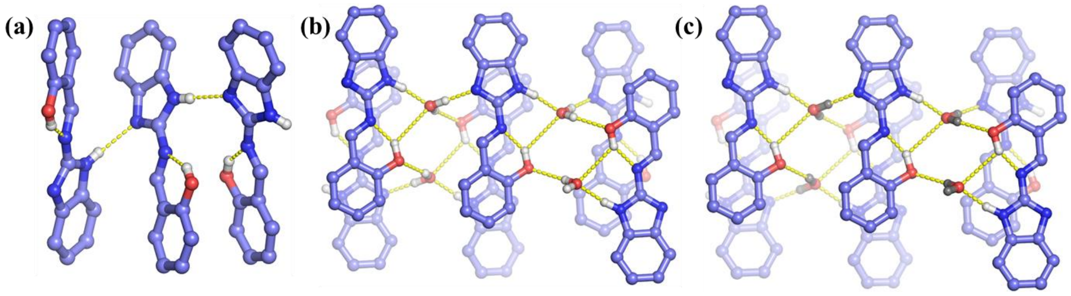

Supplementary Figure 17. Molecular stacking of prepared single crystals of (a) **1a**, (b) **1a·H$_2$O** and (c) **1b·D$_2$O** microcrystals**.** Multiple inter- and intramolecular hydrogen bonds in the single crystals were denoted by dotted lines.

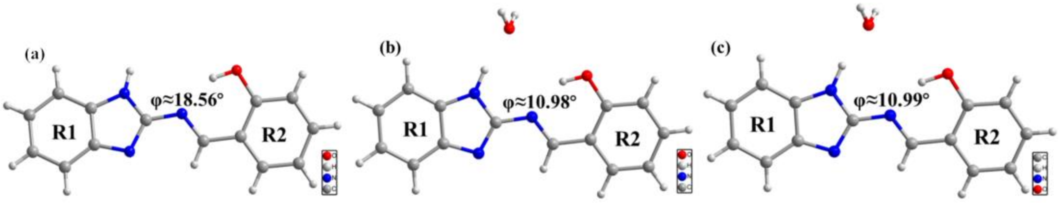

Supplementary Figure 18. Dihedral angle of molecular configuration in (a) **1a**, (b) **1a·H$_2$O** and (c) **1a·D$_2$O** single crystals.

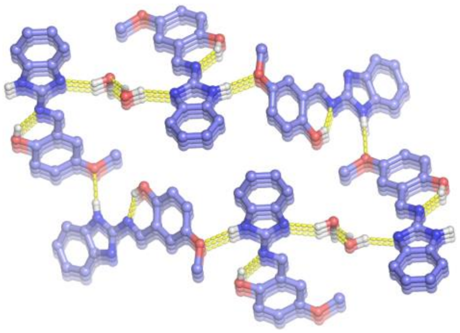

Supplementary Figure 19. Molecular stacking of prepared single crystals of **1b·D$_2$O**.

Multiple inter- and intramolecular hydrogen bonds in the crystals were denoted by dotted lines.

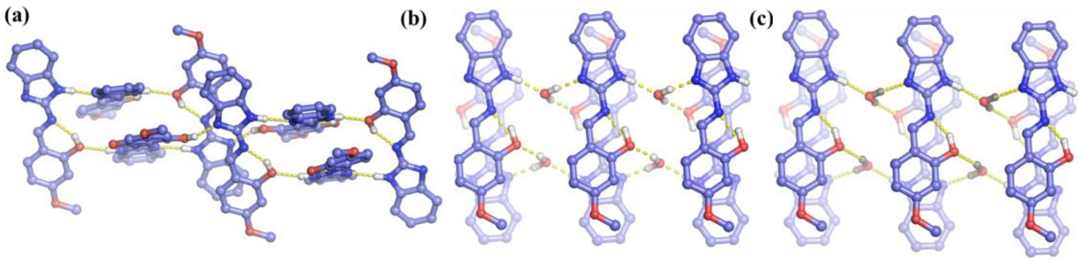

Supplementary Figure 20. Molecular stacking of prepared single crystals of (a) **1c**, (b)**1c·H_2O** and (c) **1c·D_2O**. Multiple inter- and intramolecular hydrogen bonds in the single crystals are denoted by dotted lines.

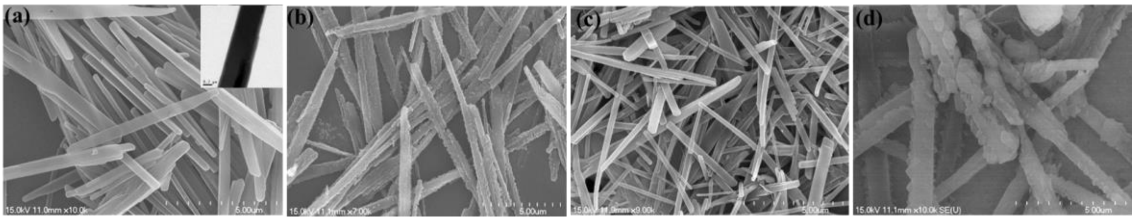

Supplementary Figure 21. Typical SEM images of: (a) **1a·H_2O** microcrystals; (c) **1a·D_2O** microcrystals**;** (b) and (d) **1a·H_2O** microcrystals and **1a·D_2O** microcrystals after heat treatment (60 ℃ for 10 min). Inset shows the magnified TEM images of **1a·H_2O** microcrystals.

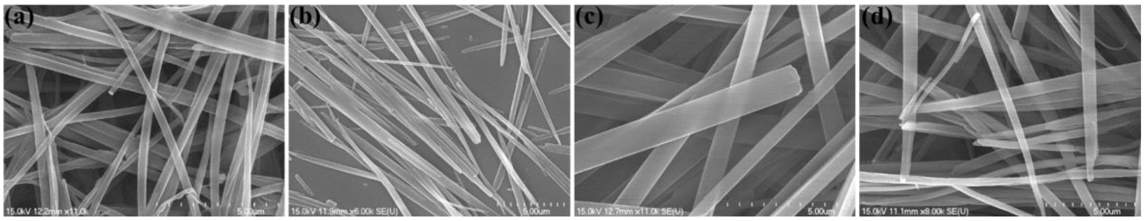

Supplementary Figure 22. Typical SEM images of: (a) **1b·H_2O** microcrystals; (c) **1b·D_2O** microcrystals**;** (b) and (d) **1b·H_2O** microcrystals and **1b·D_2O** microcrystals after heat treatment (60 ℃ for 10 mins).

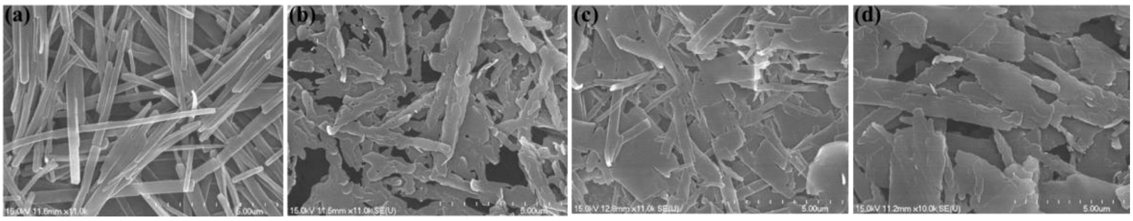

Supplementary Figure 23. Typical SEM images of: (a) **1c·H_2O** microcrystals; (c) **1c·D_2O** microcrystals; (b) and (d) **1c·H_2O** microcrystals and **1c·D_2O** microcrystals after heat treatment (60 °C for 10 min).

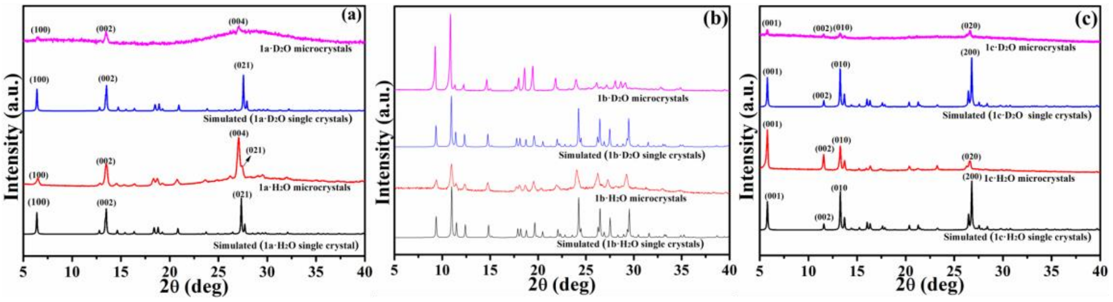

Supplementary Figure 24. X-ray diffraction (XRD) patterns of (a) **1a·H_2O (D_2O)** microcrystals, (b) **1b·H_2O (D_2O)** microcrystals and (c) **1c·H_2O (D_2O)** microcrystals based on their individual hydrated single crystals.

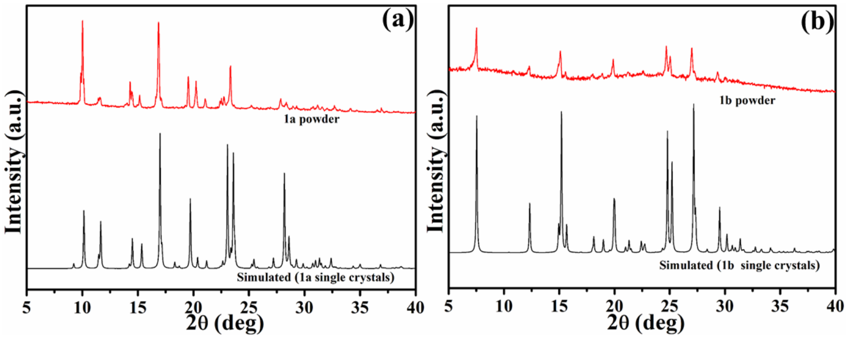

Supplementary Figure 25. X-ray diffraction (XRD) patterns of: (a) **1a** polycrystalline powder; (b) **1b** polycrystalline powder as simulated from their individual single crystals.

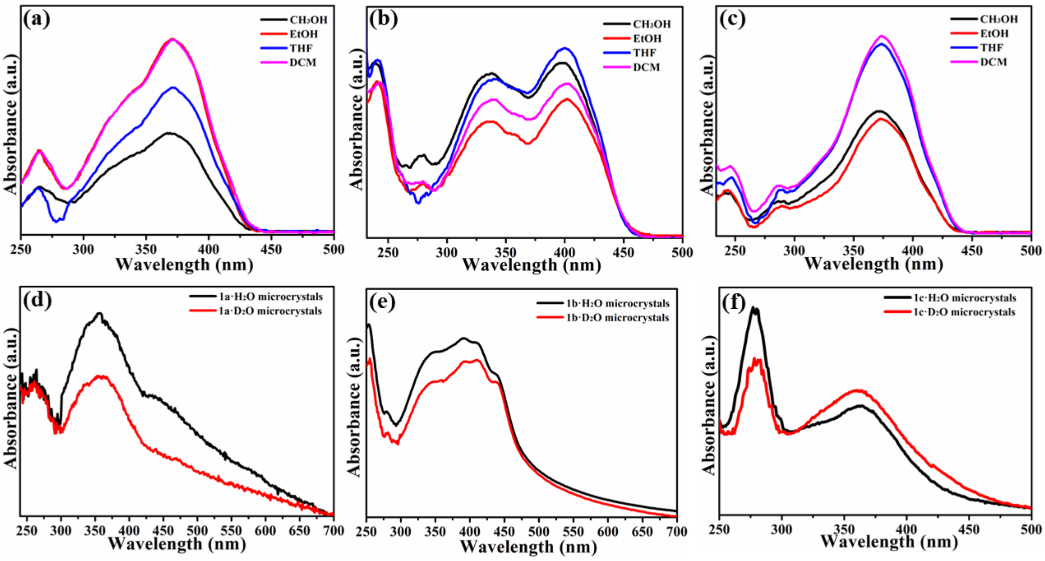

Supplementary Figure 26. UV absorption of (a) **1a**, (b) **1b**, and (c) **1c** (1×10^{-5} M) in different anhydrous organic solvents (CH_3OH, EtOH, THF and DCM). UV absorption of (d) **1a·H_2O (D_2O)** microcrystals, (e) **1b·H_2O (D_2O)** microcrystals, and (f) **1c·H_2O (D_2O)** microcrystals dispersion in water or heavy water.

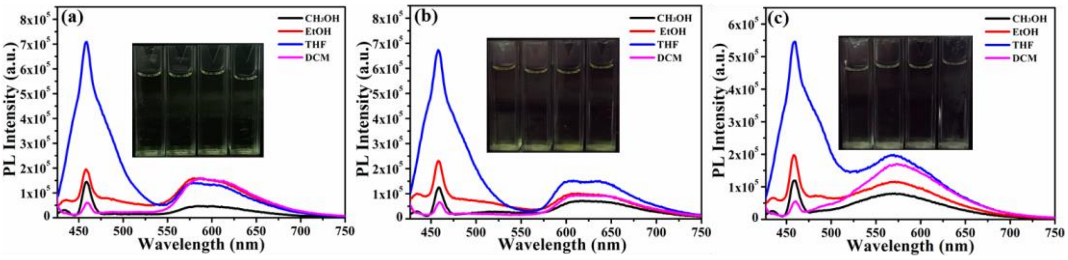

Supplementary Figure 27. Emission spectra (λ_{ex}= 400 nm) of (a) **1a**, (b) **1b** and (c) **1c** (1×10^{-5} M) in different anhydrous organic solvents (CH_3OH, EtOH, THF and DCM).

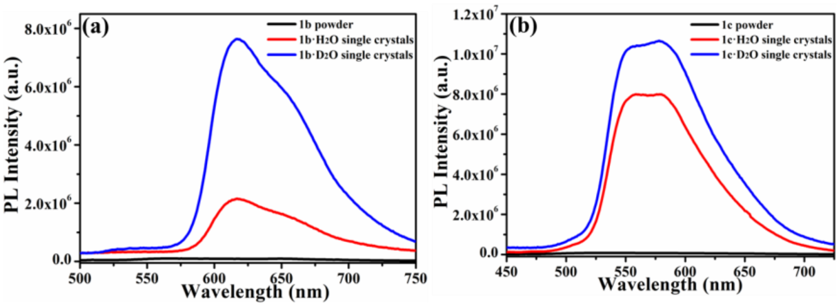

Supplementary Figure 28. Emission spectra of (a) **1b** polycrystalline powder, **1b·H_2O (D_2O)** single crystals and **1c** polycrystalline powder, **1c·H_2O (D_2O)** single crystals.

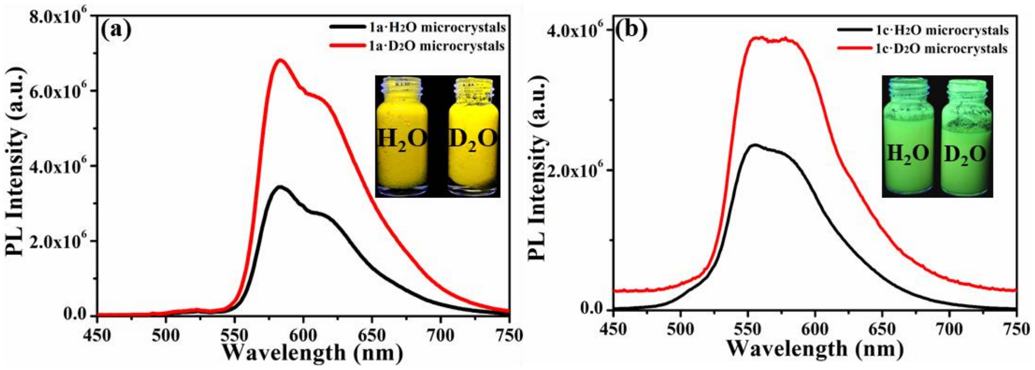

Supplementary Figure 29. Emission spectra of (a) **1a·H_2O (D_2O)** microcrystals and (b) **1c·H_2O (D_2O)** microcrystals.

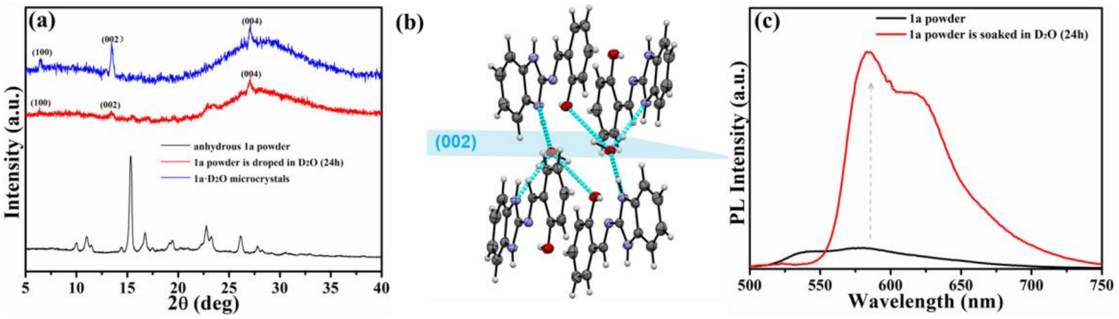

Supplementary Figure 30. (a) X-ray diffraction (XRD) patterns of hydrated **1a·H_2O (D_2O)** microcrystals based on their individual hydrated single crystals; (b) Molecular arrangement in the (002) lattice direction of **1a·D_2O** single crystals; (c) In-situ emission spectra of anhydrous 1a polycrystalline powder soaked in D_2O.

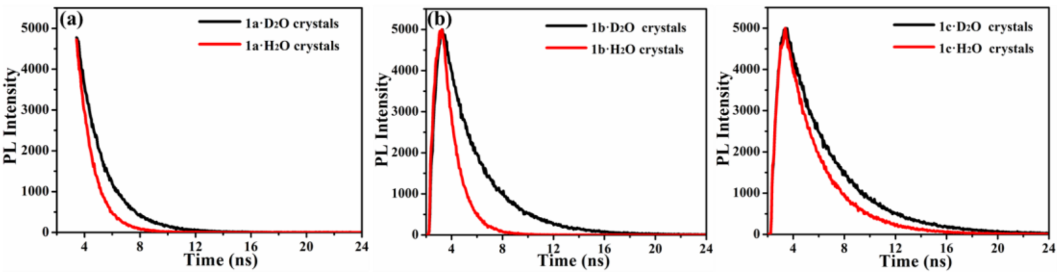

Supplementary Figure 31. Fluorescence decay curves associated with lamp profile for **1a·H_2O (1a·D_2O)**, **1b·H_2O (1a·D_2O)** and **1c·H_2O (1c·D_2O)** crystals in ambient environment. The fluorescence decay of its anhydrous crystals is too fast to be detected. Excitation wavelength is 360 nm.

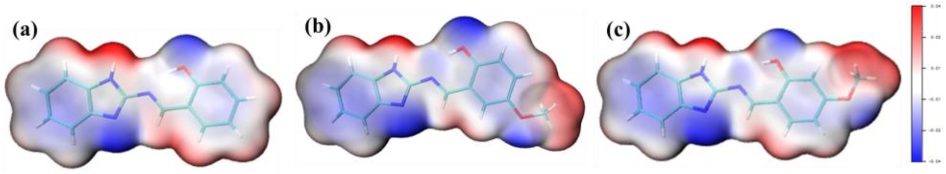

Supplementary Figure 32. Electrostatic diagram of (a) **1a**, (b) **1b** and **(c) 1c**.

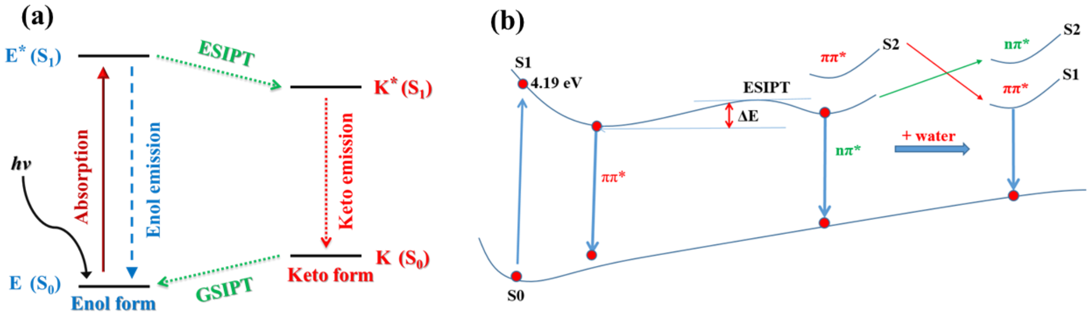

Supplementary Figure 33. (a) Four level ESIPT process; (b) Proposed activation emission mechanism for **1a·H_2O**.

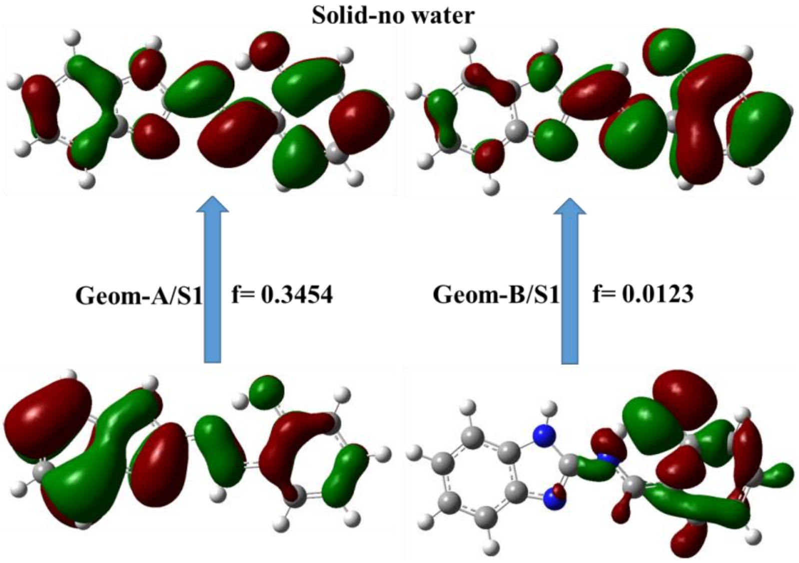

Supplementary Figure 34. Natural transition orbital (NTO) of **1a** in K* (S1) state.

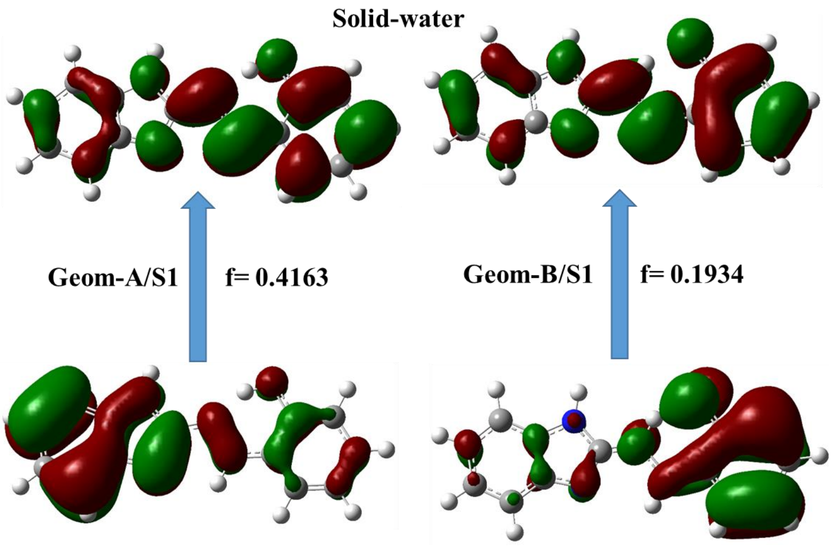

Supplementary Figure 35. Natural transition orbital (NTO) of **1a·H_2O** in K* (S1) state.

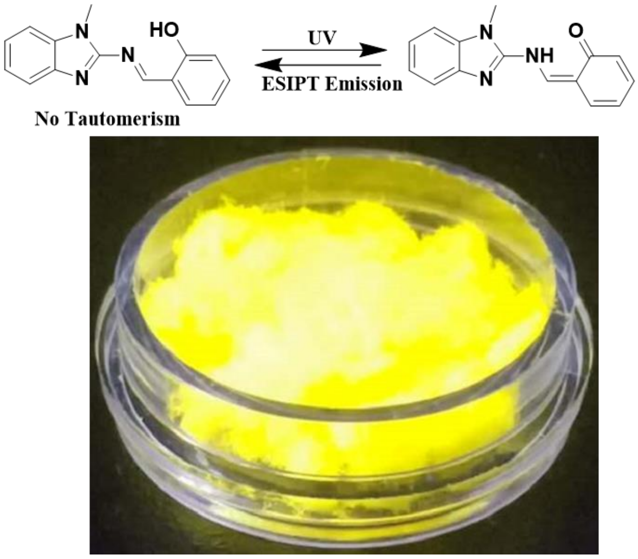

Supplementary Figure 36. **1d** in solid state has strong fluorescence due to lack of active hydrogen atom on N atoms of the benzimidazole ring.

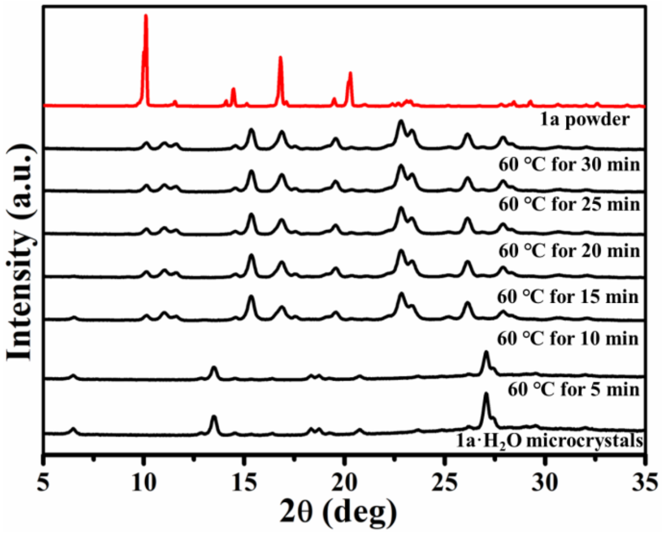

Supplementary Figure 37. XRD patterns: **1a** polycrystalline powder (red), **1a·H2O** microcrystals (black) and **1a·H2O** microcrystals after heating (black) for different times.

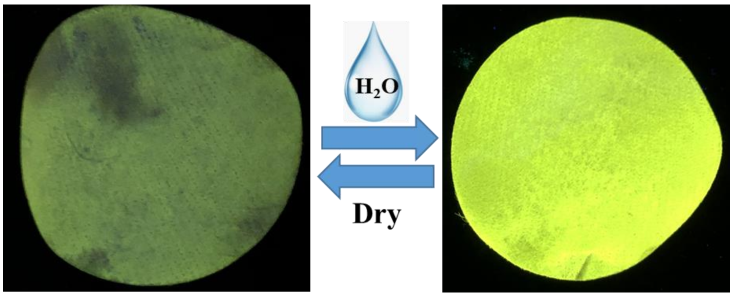

Supplementary Figure 38. Switching the luminescence of **1a** film between two processes: placing in water and heating for 60 °C for 3 mins.

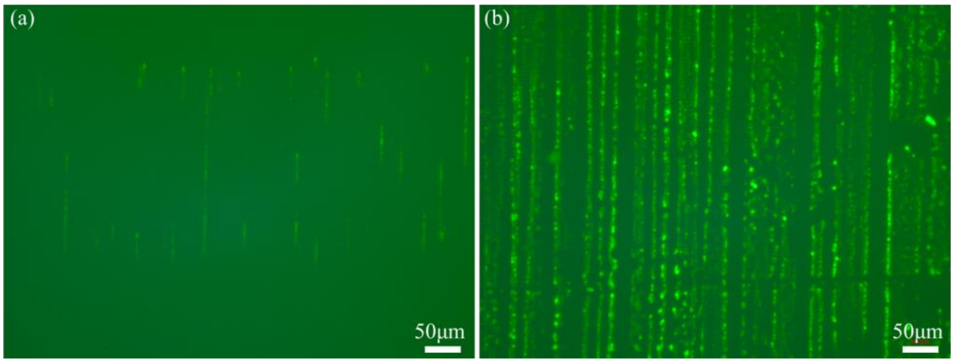

Supplementary Figure 39. Fluorescent images of **1a·H2O** (a) and **1a·D2O** (b) microcrystals grown on silicon dioxide substrate.

Supplementary Table 1. Crystallographic data for **1a, 1a·H_2O** and **1a·D_2O** single crystals.

Compound	**1a**	**1a·H_2O**	**1a·D_2O**
Formula	$C_{14}H_{11}N_3O$	$C_{14}H_{13}N_3O_2$	$C_{14}H_{11}D_2N_3O_2$
F_w	237.26	255.27	257.27
T (K)	296.15	273.15	120 K
Crystal system	Orthorhombic	Monoclinic	Monoclinic
Space group	Pnna	$P2_1/c$	$P2_1/c$
a (Å)	12.435(2)	14.2981 (14)	14.2706 (9)
b (Å)	15.411(2)	6.7302 (7)	6.6759 (4)
c (Å)	12.202(2)	13.5479 (12)	13.5114 (8)
α (deg)	90	90	90
β (deg)	90	104.647 (3)	104.580
γ (deg)	90	90	90
V (Å ^{3})	2338.3 (7)	1261.3 (2)	1245.77 (13)
Z	8	4	4
D_{calc} (Mg/m^3)	1.348	1.344	1.361
μ (mm^{-1})	0.089	0.093	0.094
F(000)	992.0	536	536.0
Independent reflections	2398	2478	2279
R (I>2σ(I))	0.1226	0.0501	0.0456
wR_2 (all data)	0.1408	0.1498	0.1517
GOF on F^2	0.993	0.996	1.204

Supplementary Table 2. Crystallographic data for **1b**, **1b·H_2O** and **1b·D_2O** single crystals.

Compound	**1b**	**1b·H_2O**	**1b·D_2O**
Formula	$C_{15}H_{13}N_3O_2$	$C_{15}H_{15}N_3O_3$	$C_{15}H_{13}D_2N_3O_3$
F_w	267.28	285.18	285.18
T (K)	294.18(10)	100	100
Crystal system	monoclinic	orthorhombic	orthorhombic
Space group	$P2_1/c$	$P\ 2_1\ 2_1\ 2_1$	$P\ 2_1\ 2_1\ 2_1$
a (Å)	5.9274(4)	4.6500(9)	4.6400(9)
b (Å)	23.4465(14)	15.400(3)	15.510(3)
c (Å)	9.2341(6)	18.880(4)	18.920(4)
α (deg)	90.00	90	90
β (deg)	100.818(7)	90	90
γ (deg)	90.00	90	90
V (Å ^{3})	1260.52(14)	1352.0(5)	1361.6(5)
Z	4	4	4
D_{calc} (Mg/m^3)	1.408	1.401	1.391
μ (mm^{-1})	0.097	0.020	0.020
F(000)	560.0	2011	2011
Independent reflections	2583	4384	5287
R (I>2σ(I))	0.1374	0.2048	0.2421
wR_2 (all data)	0.1571	0.4806	0.4750
GOF on F^2	1.046	1.951	1.586

Supplementary Table 3. Crystallographic data for **1c**, **1c·H_2O** and **1c·D_2O** single crystals.

Compound	**1c**	**1c·H_2O**	**1c·D_2O**
Formula	$C_{15}H_{13}N_3O_3$	$C_{15}H_{15}N_3O_3$	$C_{15}H_{13}D_2N_3O_3$
F_w	267.28	285.30	285.30
T (K)	120	221.91(10)	293.37(10)
Crystal system	monoclinic	triclinic	triclinic
Space group	$P2_1/c$	P-1	P-1
a (Å)	18.3220 (13)	6.9147(7)	6.9169(10)
b (Å)	14.7544 (3)	6.9980(6)	7.0078(10)
c (Å)	28.974 (6)	15.4217(15)	15.434(2)
α (deg)	90	81.740(8)	81.711(12)
β (deg)	100 . 117 (3)	88.850(8)	88.822(12)
γ (deg)	90	74.125(9)	74.053(12)
V (Å ^{3})	7710.9 (3)	710.19(12)	711.66(18)
Z	24	2	2
D_{calc} (Mg/m^3)	1.381	1.334	1.331
μ (mm^{-1})	0.496	0.095	0.095
F(000)	3360.0	300.0	300.0
Independent reflections	14149	2894	2904
R (I>2σ(I))	0.0681	0.1375	0.1449
wR_2 (all data)	0.1983	0.1738	0.1941
GOF on F^2	1.025	0.940	0.934

Supplementary Table 4. Quantum yield of **1a**, **1b** and **1c** in different organic solvents.

	**1a**	**1b**	**1c**
**CH_3OH**	0.21 %	0.24%	0.15%
**CH_3CH_2OH**	0.28 %	0.41%	0.22%
**THF**	0.39 %	0.55%	0.32%
**DCM**	0.25%	0.29%	0.19%

Supplementary Table 5. Quantum yield of hydrated BIMPs dispersed in water.

	**1a**	**1b**	**1c**
**H_2O**	15.3 %	6.3%	10.2%
**D_2O**	25.8%	29.1%	18.4%
**powder**	0.05%	0.12%	0.19%

*Fluorescence quantum yields (Φ_f) were determined with excitation wavelength at 360 nm, using fluorescein as the standard reference (Φ_f=0.79).

Supplementary Table 6. Fluorescence decay data for crystals.

**Sample**	**f_1 (%)**	**τ_1 (ns)**	**f_2 (%)**	**τ_2 (ns)**	**τ (ns)**
**1a·D_2O**	16.969	1.387	83.031	1.981	1.88
**1a·H_2O**	99.685	1.13	0.315	9.214	1.16
**1b·D_2O**	6.659	1.279	93.341	3.115	2.99
**1b·H_2O**	99.623	1.141	0.377	81.272	1.64
**1c·D_2O**	19.853	2.916	80.147	3.992	3.78
**1c·H_2O**	6.082	1.647	93.918	2.887	2.81

Supplementary Table 7. Intramolecular hydrogen bonds (N···O) in the single crystals.

	**1a**	**1a·H_2O (1a·D_2O)**	**1b**	**1b·H_2O (1b·D_2O)**	**1c**	**1c·H_2O (1c·D_2O)**
**N···O**	2.63 Å	2.56 Å (2.58 Å)	2.62 Å	2.56 Å (2.58 Å)	2.64 Å	2.61 Å (2.62 Å)

Supplementary Table 8. Intermolecular hydrogen bonds (N···O) in the crystals.

	**1a·H_2O (D_2O)**	**1b·H_2O (D_2O)**	**1c·H_2O (D_2O)**
**N···O**	2.78 Å (2.78 Å)	2.94 Å (2.95 Å)	2.79 Å (2.80 Å)

Supplementary Table 9. Radiation rate (kr~E^2f) of **1a** in different states.

	**Geom-A/S1**	**Geom-B/S1**			**Geom-B/S2**	**Fluo.**
	f	*f*	λg (eV)	λe (eV)	*f*	
1b solution	0.3821	0.0000	0.311	0.295	0.2733	weak
1b powder	0.3454	0.0123	-	-	0.2550	weak
1b·H_2O	0.4163	0.1934	0.309	0.223	0.0149	✔

Supplemenatry Reference

1. (a) Feike, M.; Graf, R.; Schnell, I.; Jager, C.; Spiess, H. Structure of¨ crystalline phosphates from ^{31}P double-quantum NMR spectroscopy. J. Am. Chem. Soc. 1996, 118, 9631-9634. (b) Saalwachter, K.; Lange, F.; Matyjaszewski, K.; Huang, C. F.;˙Graf, R. BaBa-xy16: Robust and broadband homonuclear DQ recoupling for applications in rigid and soft solids up to the highest MAS frequencies. *J. Magn. Reson.* **212**, 204-215 (2011).
2. (a) Hayashi, S.; Hayamizu, K. Chemical shift standards in highresolution solid-state NMR (1) 13C, 29Si, and 1H nuclei. Bull. Chem. Soc. Jpn. 1991, 64, 685-687. (b) Morcombe, C. R.; Zilm, K. W. Chemical shift referencing in MAS solid state NMR. *J. Magn. Reson.* **162**, 479-486 (2003).
